# Naphthoquinone-Amino Acids Regulate Cellular Cancer Associated Processes, p53 and miR-34a-5p Expression in Immortal and Tumorigenic Cervical Cell Lines

**DOI:** 10.3390/ijms27135703

**Published:** 2026-06-24

**Authors:** Jessica Lizbeth Sifuentes-Padilla, Angelica Judith Granados-López, Antonia Monserrat Campos-Lujan, Abel Suárez-Castro, Mayra Denise Herrera, Yamilé López-Hernández, Hiram Hernández-López, José Antonio Varela-Silva, Rosalinda Gutiérrez-Hernández, Claudia Araceli Reyes-Estrada, Sergio Hugo Sánchez-Rodríguez, Ernesto Rivera-Ávalos, Denisse de Loera, Jesús Adrián López

**Affiliations:** 1 Laboratorio de microRNAs y Cáncer, Unidad Académica de Ciencias Biológicas, Universidad Autónoma de Zacatecas, Av. Preparatoria S/N, Zacatecas 98068, Mexico; jsifuentespadilla@gmail.com (J.L.S.-P.); agranados@uaz.edu.mx (A.J.G.-L.); monserratcamlu@gmail.com (A.M.C.-L.); mayradherrera@gmail.com (M.D.H.); jose.varela@uaz.edu.mx (J.A.V.-S.); 2 Escuela de Medicina, Universidad Vasco de Quiroga, Av. Juan Pablo II No. 555, Col. Santa María de Guido, Morelia Michoacán 58090, Mexico; abel.suarez@umich.mx; 3 INIFAP-Campo Experimental Zacatecas (CEZAC-INIFAP), Carretera Zacatecas-Fresnillo Km 24.5, Calera de VR, Zacatecas 98500, Mexico; 4 CONACYT, Laboratorio de Metabolómica y Proteomica, Unidad Académica de Ciencias Biológicas, Universidad Autónoma de Zacatecas, Av. Preparatoria No. 301, Zacatecas 98068, Mexico; yamile@ualberta.ca; 5 The Metabolomics Innovation Center, University of Alberta, Edmonton, AB T6G 2E9, Canada; 6 Laboratorio de Síntesis Orgánica, Unidad Académica de Ciencias Químicas, Siglo XXI Campus, Universidad Autónoma de Zacatecas, Kilometer 6, Ejido la Escondida, Zacatecas 98160, Mexico; hiram.hernandez.lopez@uaz.edu.mx; 7 Laboratorio de Nutrición y Etnofarcacología, Unidad Académica de Nutrición, Siglo XXI Campus, Universidad Autónoma de Zacatecas, Kilometer 6, Ejido la Escondida, Zacatecas 98160, Mexico; rosalindagh@hotmail.com; 8 Laboratorio de Immunohistoquímica y Estrés Oxidativo, Maestría en Ciencias de la Unidad Académica de Medicina Humana, Siglo XXI Campus, Universidad Autónoma de Zacatecas, Kilometer 6, Ejido la Escondida, Zacatecas 98160, Mexico; c_reyes13@yahoo.com.mx; 9 Laboratorio de Biología Celular y Neurobiología, Unidad Académica de Ciencias Biológicas, Universidad Autónoma de Zacatecas, Zacatecas 98068, Mexico; smdck@uaz.edu.mx; 10 Secretaria de Ciencias, Humanidades, Tecnología e Innovación (SECIHTI), Laboratorio de Cromatografía y Absorción Atómica, Laboratorio Estatal de Salud Pública del Estado de Zacatecas, Secretaria de Salud del Estado de Zacatecas, Vialidad Arroyo de la Plata 1, Zona Industrial, Guadalupe, Zacatecas 98600, Mexico; ernesto.rivera@secihti.mx; 11 Escuela de Química, Universidad Autónoma de San Luis Potosí, Av. Manuel Nava No. 6, San Luis Potosí 78210, Mexico; atenea.deloera@uaslp.mx

**Keywords:** cervical cancer, p53, miR-34a-5p, tumorigenic cells, immortal cells

## Abstract

Cervical cancer is a malignant disease that affects women worldwide and is associated with both high incidence and a high mortality rate. miR-34 is a direct transcriptional-target of p53 and is downregulated in several types of cancers. 1,4-Naphthoquinones (NQs) have anticancer properties and have been used to modulate miR-34 expression. We tested (3-chloro-NQ-2-yl)-alanine (ANQCl), -methionine (MNQCl), -glycine (GNQCl), -phenylalanine (FNQCl), -asparagine (NNQCl), and (1,4-napthoquinon-2-yl)-asparagine (NNQ) in immortal and tumorigenic cells, both HPV-positive and -negative, simulating precancerous and cancerous status to observe the response of the p53-miR-34 system, migration and invasion. A dose–response was achieved to determine the IC50 of the compounds in SiHa, CaLo, C33-A and HaCaT cells. HaCaT cell migration inhibition was more potent than in SiHa, CaLo, and C33-A cells, while invasion hindrance was more evident in the tumorigenic SiHa, CaLo and C33-A. NNQCl, GNQCl, ANQCl and FNQCl compounds induced p53 overexpression in SiHa and CaLo cells. Compound ANQCl in SiHa and FNQCl in CaLo induced miR-34a overexpression, probably via p53. Migration and invasion of most compounds decreased independently of p53-miR-34. NQ-amino acids exert effect on cell proliferation, migration and invasion in cervical cancer cells, suggesting their potential use in the field of cancer treatment.

## 1. Introduction

Cervical cancer is the second most common cancer in women worldwide, with an estimated global incidence of 470,000 new cases and more than 200,000 deaths per year [1]. High-risk human papillomavirus (HPV) infection has been associated with the development of cervical, penile, and anal cancers. Although high-risk HPV expression appears necessary to immortalize and stimulate proliferation in human keratinocytes [2], it is not sufficient to produce a fully transformed phenotype, suggesting that additional genetic changes participate in the development of cervical cancer, among other risk factors [3]. In high-risk HPV-infected cells, interference with p53 and retinoblastoma proteins (pRB) functions via the viral oncoproteins E6 and E7 disrupts the expression of hundreds of genes associated with cell-cycle regulation, DNA repair, and apoptosis. The overall effect of E6/E7 expression is reflected in genomic instability, unrestricted E2F bypass of the gap 1–synthesis (G1–S) checkpoint, and inhibition of p53-mediated functions, including apoptosis [4,5,6,7,8]. The miR-34 family comprises three evolutionarily conserved miRNAs: miR-34a, miR34b, and miR-34c expressing 5p strands (guide strand) and 3p strands or strand* (passenger strand). All three are described as direct targets of p53 and possess anti-proliferative and pro-apoptotic functions [9,10,11,12]. Specifically, miR-34a is the best-characterized family member and is located within exon 2 of its primary transcript, while miR-34b and miR-34c are produced from a different locus and positioned within intron 1 and exon 2 of the single primary transcript, respectively. In addition to the seed miR-34 sequences themselves, the only other region of significant sequence conservation in miR-34 genes lies in their putative promoter regions harboring p53-binding sites [11]. Since the high-risk HPV E6 oncoprotein destabilizes p53, downregulated expression of the whole miR-34 family may be expected in HPV-positive cells. It has been demonstrated that miR-34a is downregulated by E6 expression and the overexpression of miR-34a induces cell-cycle arrest and inhibits proliferation in cervical carcinoma cells [13].

Each member of the miR-34 family has two identified mature miRNAs: miR-34a-5p, miR-34a-3p, miR-34b-5p, miR-34b-3p, miR-34c-5p, and miR-34c-3p [14,15]. The 5p strands of miR-34a, b, and c are involved in proliferation halting, anchorage-independent growth, migration, and invasion, and apoptosis induction in a variety of models; therefore, they are currently considered as tumor suppressors [16,17,18,19,20]. During the search for anticancer approaches, NQs have been produced and tested with promising results. NQs are a group of natural and synthetic compounds that are known for their antibacterial, antifungal, anticancer, antimalarial, and antiviral effects, among others [21,22]. Interestingly, the addition of different types of substituents to the naphthoquinone structure, including paclitaxel, esters, metals, furans, carbazoles, or carbohydrates, among others, can impede cell proliferation of tumorigenic cell lines [23,24,25]. The properties of these compounds have been principally attributed to the oxidant-reductive characteristics of NQs, which allow the generation of dianions or semiquinone radicals. One of the principal effects of these compounds is the generation of reactive oxygen species (ROS), which produce cytotoxicity in different cell lines [26,27,28,29,30,31]. ROS generation with NQs represents a challenge in the design of new active compounds, principally with an anticancer effect. In this context, the addition/substitution of atoms or groups such as flour, oxygen, or amine on NQ moiety can modulate their redox properties, leading to decreased toxicity levels and also maintaining or potentiating the biological effect [32,33,34].

Cell proliferation inhibition through NQs has been shown to be achieved by distinct mechanisms such as the induction of apoptosis, topoisomerase II-α inhibition, and ROS generation, among others [24]. Furthermore, it has been shown that NQ β-lapachone in particular can inhibit epidermal growth factor receptor (pEGFR), protein kinase B (pAKT/PKB), glycogen synthase kinase (pGsk-3β), cyclin D1, and cyclooxygenase-2 (COX-2) protein expression in a dose-dependent manner [35]. It is therefore noticeable that NQ-dependent regulation relies on the composition of its substituents, as well as cellular targets.

Motivated by the necessity of discovering new compounds with favorable effects on cancer cells, previously, our research group synthesized chloride and non-chloride NQ amino acid derivatives and studied their potential antitumorigenic activity against cervical cell lines with distinct tumorigenic capabilities [36]. Generally, the compounds elicited 90 to 40% of proliferation inhibition of cervical cancer cell lines; interestingly, chloride NQ amino acid derivatives exhibited stronger effect than non-chloride compounds [37]. Therefore, herein, we tested ANQCl, MNQCl, GNQCl, FNQCl, NNQCl, and NNQ, naphthoquinones in SiHa (Tumorigenic and HPV16 positive), CaLo (Tumorigenic and HPV18 positive), C-33A (Tumorigenic and HPV negative) and HaCaT (Keratinocytes immortal HPV negative) cell lines in immortal and tumorigenic cells to observe the response of the p53-miR-34 system, migration and invasion in different cellular contexts simulating precancerous and cancerous status.

## 2. Results

### 2.1. (3-Chloro-1,4-naphthoquinon-2-yl)-alanine, -methionine, -glycine, -phenylalanine, -asparagine, and (1,4-Naphthoquinon-2-yl)-asparagine Have Antioxidant Capacity

**Scheme 1:**
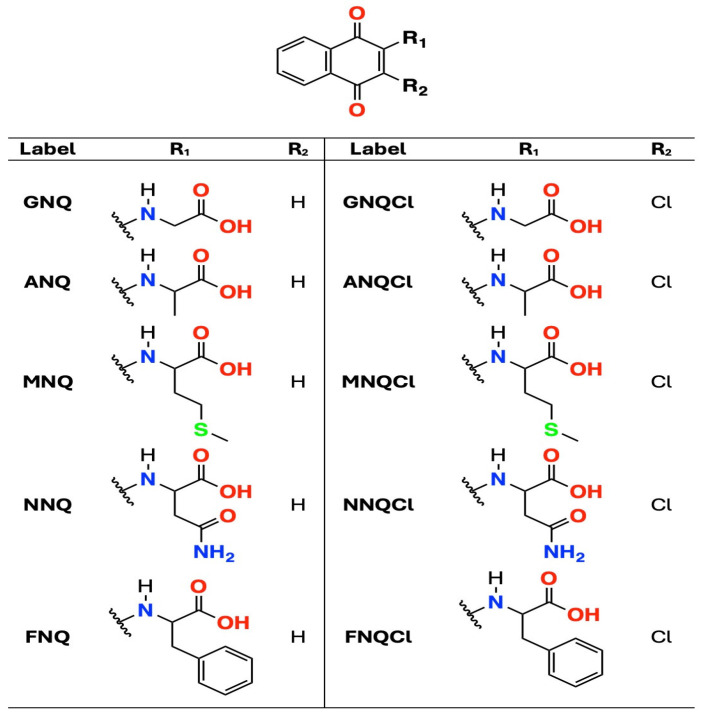
Chemical structure of NQ-amino acids. In the upper part of the scheme, a general structure of NQ is shown and in the lower part, the substituent radicals 1 and 2 are shown.

**Figure 1:**
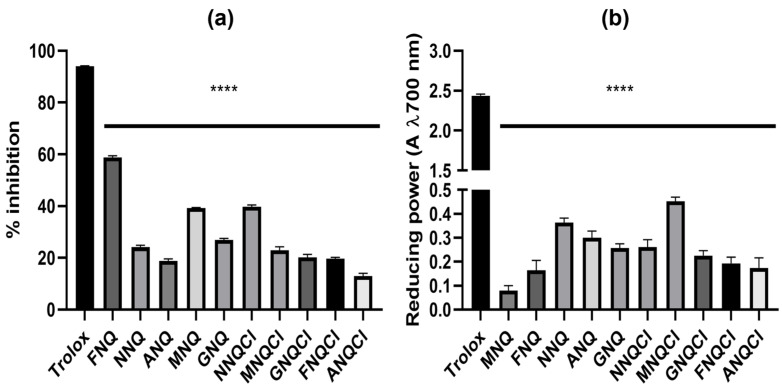
In vitro antioxidant capacity of NQ-amino acids with or without chlorine atom. (**a**) NQs exhibit ABTS scavenging capacity and (**b**) reducing power. The antioxidant capacity of the NQs was assessed to determine the capacity to scavenge the free radical ABTS. Each NQ was diluted with DMSO and the radical solution was added. The absorbance of NQs and a control was measured. The data correspond to three replicates and three independent experiments (**** *p* ≤ 0.0001).

Cancer cells exhibit elevated levels of both intracellular and extracellular reactive oxygen species (ROS) that cause oxidative stress. The enhanced level of intracellular ROS is due to inadequate production or detoxification mechanisms that contribute to cells being transformed into more malignant phenotypes [38]. Since oxidative stress has been associated with cervical cancer, the NQ-amino acids with or without chlorine atoms bonded in C-3 of NQ moiety (Scheme 1) were analyzed to screen their antioxidant capacity in vitro (Figure 1). Although compound FNQ had the highest capacity to scavenge the ABTS free radical, followed by NNQCl and MNQ (Figure 1a), it is worth mentioning that all tested compounds exhibited antioxidant rather than pro-oxidant properties. NQs may undergo redox cycling, in some cases developing both an antioxidant or pro-oxidant response, depending on the biological environment [39]. Likewise, all NQs tested exhibited reducing power; however, MNQCl compound followed by NNQ displayed a significantly stronger reducing effect (Figure 1b). Subsequently, cell proliferation in NQ response was evaluated to determine whether oxidation activity correlated with cell proliferation inhibition.

### 2.2. (3-Chloro-1,4-naphthoquinon-2-yl)-alanine, -methionine, -glycine, -phenylalanine, -asparagine, and (1,4-Naphthoquinon-2-yl)-asparagine Inhibit Cervical Cancer Cell Proliferation

SiHa, C-33A, CaLo and HaCaT cells were treated with different doses of non-chloride and chloride NQ-amino acids to determine IC50 (Table 1). NNQ and NNQCl exerted a stronger effect in tumorigenic cells than in immortal cells. While the former preferentially inhibited SiHa, the second was strongest in C-33A cells. In the same manner, ANQCl, MNQCl, GNQCl and FNQCl specifically inhibited the C-33A cell line. With the exception of FNQCl and GNQCl, the rest of the compounds elicited a stronger proliferation repression effect on tumorigenic than on immortal HaCaT cells. HPV content appears to be irrelevant to the NQ action, as implied by similar effects observed in C-33A HPV- and CaLo HPV+ cells. NQ actions may exert different cellular processes responses; therefore, we sought to analyze the effect of the non-chloride and chloride NQs on cell migration.

### 2.3. (3-Chloro-1,4-naphthoquinon-2-yl)-alanine, -methionine, -glycine, -phenylalanine, -asparagine, and (1,4-Naphthoquinon-2-yl)-asparagine Inhibit Cervical Cancer Cell Migration

**Figure 2:**
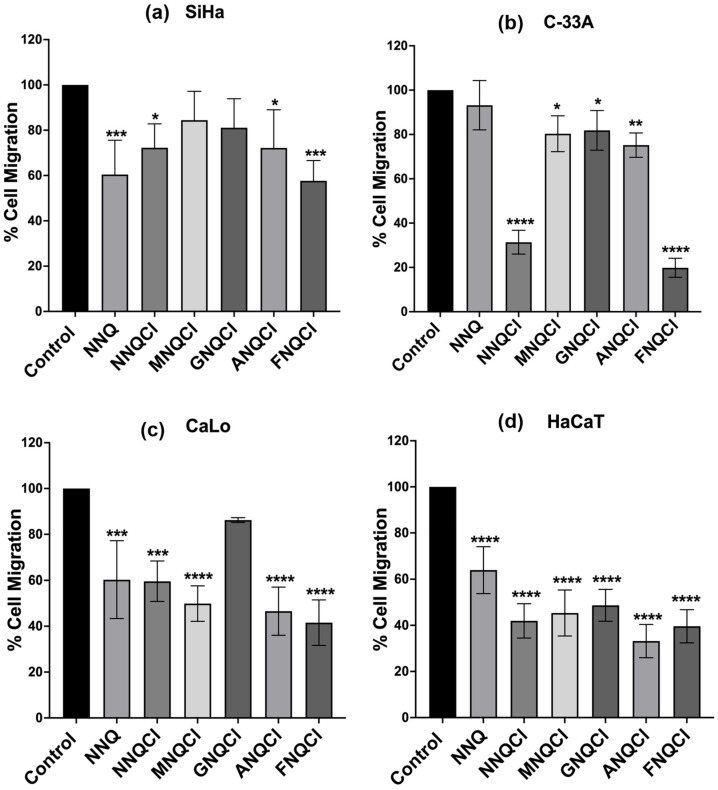
Treatment with naphthoquinone-amino acids inhibits migration of SiHa, C-33A, CaLo and HaCaT cells. The cells (**a**) SiHa, (**b**) C-33A, (**c**) CaLo, and (**d**) HaCaT were treated with the IC50 of NQ-amino acids for 24 h, subsequently, were washed and counted and 80,000 cells were seeded and incubated for 72 h using 8 µm inserts. The data correspond to three replicates and three independent experiments. The results are presented as the mean ± SD. Comparisons were performed by an analysis of variance test (ANOVA) with a Dunnet’s post hoc test. It was considered statistically significant when *p* ≤ 0.05. * *p* = 0.0332, ** *p* = 0.0021, *** *p* = 0.0002, **** *p* ≤ 0.0001.

SiHa, C-33A, CaLo and HaCaT cells were treated with their corresponding IC50 of non-chloride and chloride naphthoquinone-amino acids to determine cell migration inhibition. In SiHa cells, the NNQCl and FNQCl inhibited approximately 40% of cell migration, while ANQCl and NNQCl blocked 30% of the process. MNQCl and MNQ did not provoke a change in this activity (Figure 2a). In C-33A cells, the non-chloride naphthoquinone was the only compound that did not exert an effect on cell migration, while ANQCl, MNQCl and GNQCl exerted an inhibitory effect near 20%, and NNQ and FNQCl had a repressive effect around 75% (Figure 2b). Interestingly, in CaLo cells, a notable effect on cell migration was achieved compared to the effect in cell proliferation, which presented the least significant effect of all tested tumorigenic cells. ANQCl, MNQCl and FNQCl repressed almost 55% of cell migration while NNQ and NNQCl suppressed 40% (Figure 2c). Interestingly, cell migration was recorded with greater potential in the immortal HaCaT cell. The chloride compounds arrested migration at close to 65%, while the NNQ halted 40% of the process (Figure 2d). Several of the tested naphthoquinones inhibited cell migration; therefore, we analyzed the invasion capability of cells exposed to the compounds as a complementary cell process in metastasis.

### 2.4. (3-Chloro-1,4-naphthoquinon-2-yl)-alanine, -methionine, -glycine, -phenylalanine, -asparagine, and (1,4-Naphthoquinon-2-yl)-asparagine Inhibit Cervical Cancer Cell Invasion

**Figure 3:**
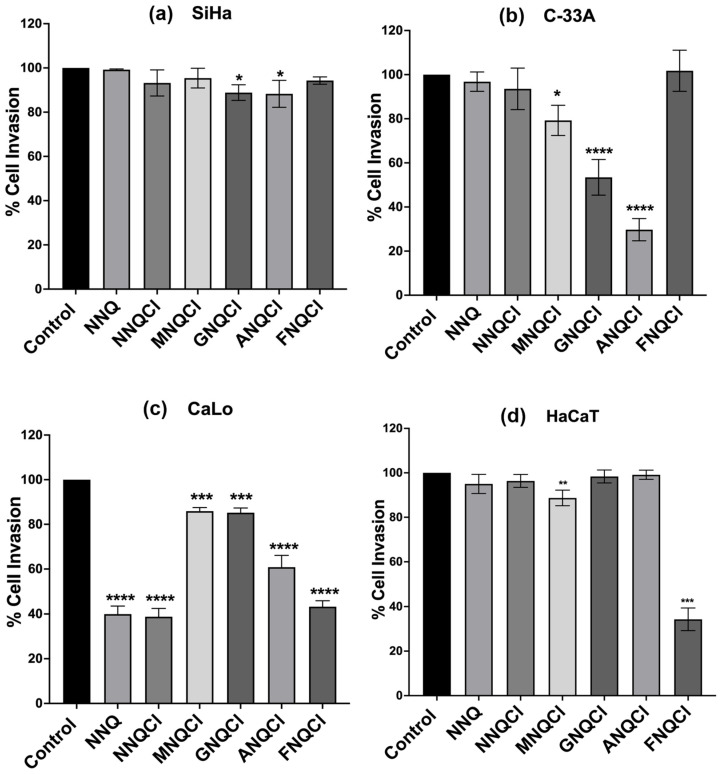
Treatment with amino acid naphthoquinones inhibits cell invasion of SiHa, C-33A, CaLo and HaCaT cells. The cells (**a**) SiHa, (**b**) C-33A, (**c**) CaLo, and (**d**) HaCaT were treated with the IC50 of NQ-amino acids for 24 h, subsequently, were washed and counted and 80,000 cells were seeded and incubated for 72 h using 8 µm inserts with matrigel. The data correspond to three replicates and three independent experiments. The results are presented as the mean ± SD. Comparisons were performed via an analysis of variance test (ANOVA) accompanied by a Dunnet’s post hoc test. It was considered statistically significant when *p* ≤ 0.05. * *p* = 0.0332, ** *p* = 0.0021, *** *p* = 0.0002, **** *p* ≤ 0.0001.

SiHa, C-33A, CaLo and HaCaT cells were treated with corresponding IC50s of non-chloride and chloride naphthoquinone-amino acids to determine cell invasion inhibition. The effect in cell invasion of SiHa cells was almost null. ANQCl and GNQCl inhibited 20%, while the rest had no effect (Figure 3a). In C-33A cells, the effect of 75%, 55% and 25% for ANQCl, GNQCl and MNQCl of cell invasion inhibition was recorded. The rest of the compounds did not exert any effect on this cell process (Figure 3b). In CaLo cells, all the compounds exerted a noticeable effect, with remarkable effects observed for NNQCl and NNQ (Figure 3c). Interestingly, in HaCaT cells, only MNQCl and FNQCl exerted an effect (Figure 3d). Several cellular processes such as proliferation, migration and invasion may be regulated under the control of different proteins. Therefore, the involvement of the p53-miR34 pathway was explored in order to inquire the mechanism of NQ’s effect on cell lines.

### 2.5. (3-Chloro-1,4-naphthoquinon-2-yl)-alanine, -methionine, -glycine, -phenylalanine, -asparagine, and (1,4-Naphthoquinon-2-yl)-asparagine Regulate p53-miR-34 System Protein Expression in Cervical Cancer Cells

**Figure 4:**
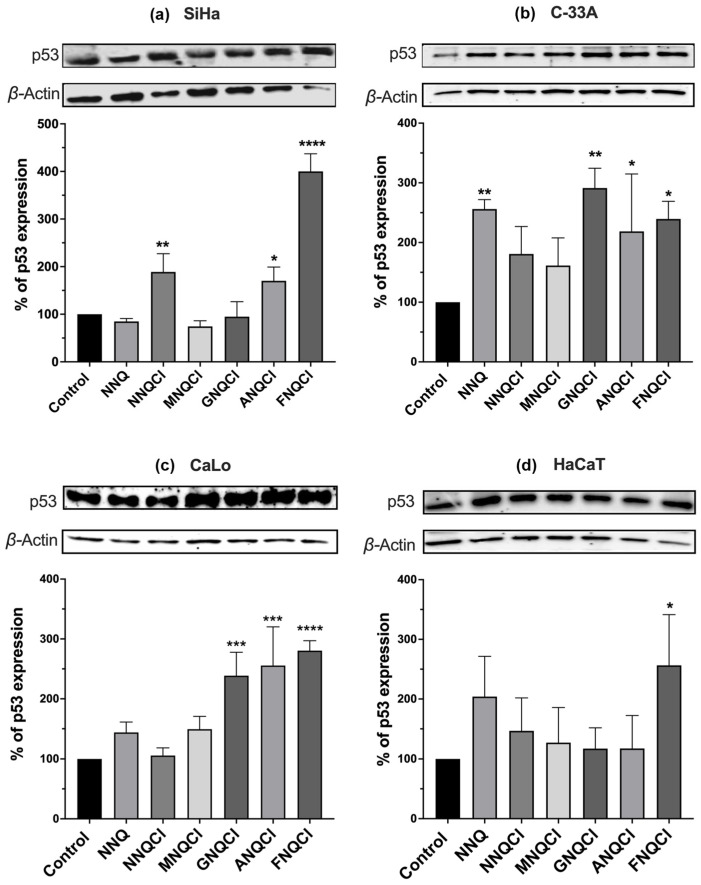

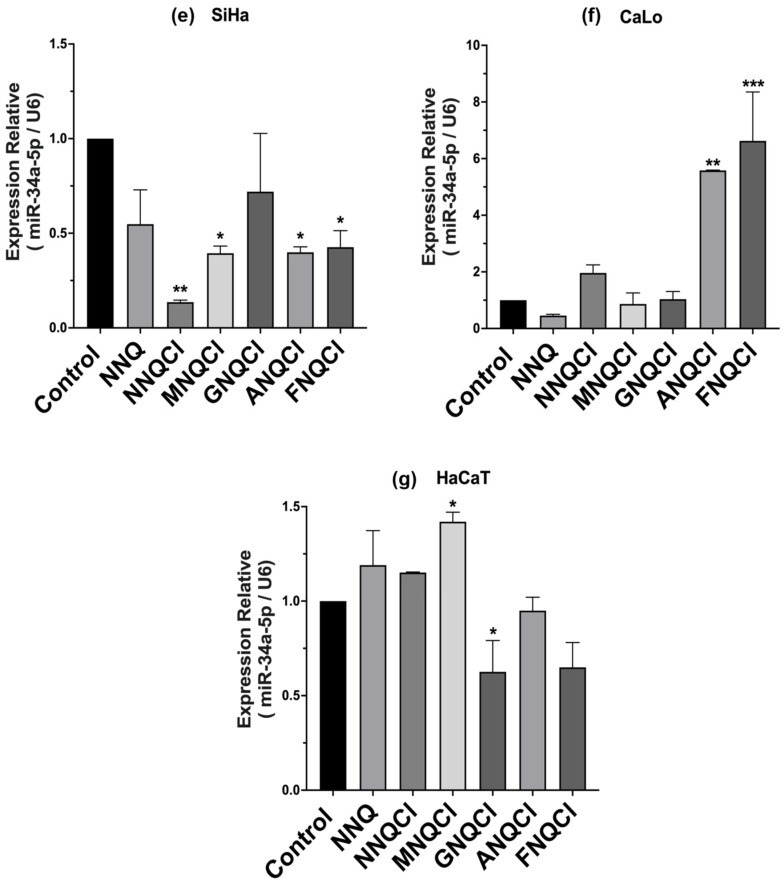
Treatment with naphthoquinone-amino acids regulates p53 and miR-34 expression in SiHa, C-33A, CaLo and HaCaT cells. The cells (**a**) SiHa, (**b**) C-33A, (**c**) CaLo, and (**d**) HaCaT were exposed to the IC50 of NQ-amino acids and protein expression was analyzed at 48 h post treatment. The graphs show the percent of p53 protein expression relative to Actin protein expression of treated cell lines. The data correspond to three replicates and three independent experiments. The results are presented as the mean ± SD. Comparisons were performed by an analysis of variance (ANOVA) test with a Dunnet’s post hoc test. It was considered statistically significant when *p* ≤ 0.05. * *p* = 0.0332, ** *p* = 0.0021, *** *p* = 0.0002, **** *p* ≤ 0.0001. The cells (**e**) SiHa, (**f**) CaLo, and (**g**) HaCaT were exposed to the IC50 of NQ-amino acids and miR-34a-5p expression was analyzed at 48 h post treatment. The graphs show miR-34a-5p expression relative to U6 expression. The data correspond to two replicates and three independent experiments. The results are presented as mean ± SD. Comparisons were performed by an analysis of variance (ANOVA) test in combination with a Dunnet’s post hoc test. *p* ≤ 0.05 was considered to be of statistical significance.

SiHa, C-33A, CaLo and HaCaT cells were treated with different IC50 amounts of non-chloride and chloride NQ to determine p53 expression. The increased expression of p53 in SiHa cells was significant with NNQCl, ANQCl and FNQCl compound treatment, while for the rest of the compounds the protein expression remained similar to that of control cells (Figure 4a). In contrast, in C-33A cells, the treatment with NNQ, GNQCl, ANQCl, FNQCl elicited a p53-augmented expression while with the treatment with the rest of NQ-amino acids, no effect was observed (Figure 4b). In CaLo cells, GNQCl, ANQCl and FNQCl treatments led to an increase in p53 protein expression (Figure 4c). Interestingly, in HaCaT cells, only FNQCl treatment exerted increased expression of p53 (Figure 4d).

In search of characterization of the p53 function, we tested the expression of miR-34a-5p in the compound-treated cell lines. In opposition to our expectations, a statistically significant decrease in miR-34a-5p in cells treated with NNQ, MNQCl, NNQCl and FNQACl, was recorded in SiHa cells (Figure 4e). In C-33A cell line, a mutation in the 273 codon, conducive to a change in arginine by cysteine of the DNA-binding domains of p53 protein, has been reported [40]. Additionally, Wang et al. reported a null expression of miR-34a-5p in these cells [13]. Therefore, as could be expected, we were unable to detect miR-34a-5p in C-33A cells. On the contrary, CaLo cells exhibited increased expression of miR-34a-5p with ANQCl and FNQCl treatment (Figure 4f). Intriguingly, in HaCaT cells, an augmented expression of miR-34a-5p was achieved in cells treated with MNQCl and diminished with NNQCl compound exposure (Figure 4g).

### 2.6. Molecular Docking of p53 and NQ

**Figure 5:**
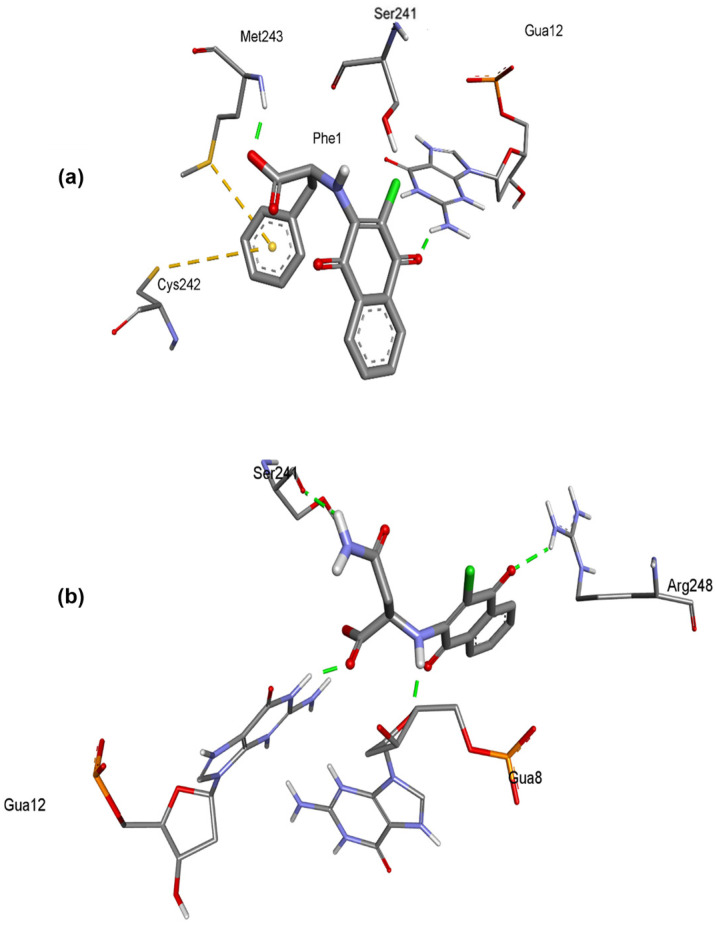
Interaction between p53 and NQ-amino acids. (**a**) Interaction between p53 and FNQCl (**b**) Interaction between p53 and NNQCl.

In light of the compounds’ effects on proliferation, migration, invasion cell processes, and p53 expression, the interaction of NQ-amino acids with p53 protein was evaluated through a molecular modeling approach. After the molecular docking experiments were performed, the 10 best poses obtained for each ligand with the p53 receptor were evaluated, and those with the lowest interaction energy were selected (Table 2). Subsequently, the main chemical interactions predicted between each ligand and the receptor were analyzed in detail, considering only the pose with the lowest interaction energy. The interaction analysis (Figure 5) was carried out using the algorithm implemented in Discovery Studio Visualizer, focusing on relevant interactions such as hydrogen bonds, pi–pi interactions, and hydrophobic interactions, among others that met the pre-established criteria.

## 3. Discussion

To date, it is uncertain if the antioxidant capacity of proposed anticancer compounds is the desired pharmacological action. While a variety of studies have demonstrated that the low level of antioxidants leads to the production of free radicals that cause lipid peroxidation, DNA, and protein damage, leading to mutations that favor malignant transformation [41,42], on the other hand it has been stated that compounds with high antioxidant capacity might protect cancer cells from oxidative damage, potentially allowing them to survive and proliferate. For instance, Sayin et al. [43] mentioned that the use of antioxidants like N-acetylcysteine and vitamin E increased tumor progression and reduced survival in lung cancer mouse models by reducing ROS, DNA damage, and p53 expression; thus, those antioxidants accelerated tumor growth by disrupting the ROS-p53 axis. However, N-acetylcysteine and vitamin E lack the characteristic naphthalene ring of naphthoquinones, which have been proven to exhibit anti-proliferative and apoptosis induction potency [44] in addition to antioxidant capacity. The antioxidant capacity of naphthoquinones is attributed to their electron-accepting capabilities forming highly reactive radicals that interact with crucial biological molecules like DNA, enzymes, and proteins, which is a central aspect contributing to their efficacy in various therapeutic applications [45]. A series of amino alcohol derivatives from 1,4-naphthoquinone were synthesized and evaluated as antioxidant and anti-proliferative agents. Interestingly, the amino-alcohol derivatives against cervix cell line HeLa exerted a 50% growth inhibition and an important antioxidant capacity relying mostly on the naphthoquinone fragment, probably due to their redox and electrophile properties, by their direct molecular action or by interacting with different enzymes which can regulate the oxidative conditions of the cells [46]. Although all the chloride and non-chloride NQ-amino acid compounds tested herein have antioxidant activity, we did not evaluate this capacity in the cell lines; therefore, their effect can be attributed to REDOX regulation or interaction with biomolecules and/or by having both characteristics.

Previously, our group showed that (3-chloro-naphthoquinone-2-yl) or (naphtoquinone-2-yl) bonded to alanine, methionine, phenylalanine, glycine, and asparagine amino acids inhibited cell proliferation ranked from ~40% to 85% and 30% to 90% in SiHa and MCF-7 cells, respectively. Notoriously, the cervix cell line, SiHa, was susceptible to inhibition by non-chloride NQs while breast cancer cell line MCF-7 was susceptible to inhibition from chloride NQ-amino acid compounds [36]. Nevertheless, this observation was weak, since it was only seen in one cell type from each type of cancer. Continuing with the compound characterization, we added HPV18- and HPV16-positive and HPV-negative cervical cancer cell lines to further clarify our previous observations with non-chloride and chloride naphthoquinone-amino acids. In general, the chloride naphthoquinone-amino acids exerted a stronger effect in the four lines tested than the non-chloride naphthoquinones, and thus we concluded that our previous observation was due to amino acids in NQ structures and not a result of the presence or absence of chloride [37]. In our previous study, glycine, asparagine, phenylalanine, alanine, and methionine naphthoquinones were tested in SiHa and MCF-7 cells [36], while the effects of tyrosine, valine, and tryptophan naphthoquinones were proven in SiHa, C-33A, CaLo, and HaCaT cell lines [37]. In the present work, of the 10 naphthoquinone-amino acids tested, NNQ, GNQCl, MNQCl, ANQCl, and FNQCl were selected based on their cell proliferation inhibition. MNQCl exerted an IC50 from 3.5 to 7.8 µM, ANQCl 3.4 to 8 µM and FNQCl 3.7 to 10.7 µM while their non-chloride NQs elicited almost five times lesser effect. GNQ and GNQCl and NNQ and NNQCl produced a similar effect; however, GNQCl and NNQ were selected for further characterization because of the weaker effect being exerted in immortal HaCaT cells than in tumorigenic cells, SiHa, CaLo and C-33A. Additionally, GNQCl doubled the effect of that exerted by GNQ in C-33A and CaLo cells. Differing effects among cell lines may result from distinct factors; for instance, cell line tissue origin and genetic background, the particular properties developed by the immortal and tumorigenic cells, and the capability of cell lines’ compound internalization and intracellular metabolism, as well the molecular targets present in the cell that may interact with the NQ-amino acids, leading to a cell-specific effect. In the present work, HPV status was irrelevant because a similar effect was recorded in the immortal and tumorigenic HPV-negative HaCaT and C-33A cells, respectively, as well as in the tumorigenic HPV 16 and 18 SiHa and CaLo cells, respectively. Importantly, considerable effects of various compounds were observed in the cell proliferation process among all cell lines. Similar proliferation inhibition was recorded in the breast cancer cell line MDAMB-435 with values of 100%, 100%, and 36%, and in glioblastoma SF-295 cells 86%, 83%, and 59% of NQ compounds [47]. A similar effect was achieved by our group with chloride phenylalanine, alanine, asparagine, and methionine naphthoquinones and non-chloride asparagine naphthoquinone. The effect of glycine, alanine, and phenylalanine derivatives synthesized by de Moraes et al. [47] was similar to our results, highlighting their potential as cytotoxicity compounds in cancer. Marastoni et al. [48] incorporated leucine, asparagine, phenylalanine and serine amino acids into naphthoquinone ring with diamine alkyl spacers inhibiting three subunits of proteasome linked to proliferation inhibition of the breast cancer cell line MDA and ovarian cancer cell line A2780 in the range of 100 μM. Remarkably, it should be noted that the chloride naphthoquinone-amino acids achieved a similar effect with a concentration that was 10 times lower. Additionally, in Marastoni et al.’s work, the compounds’ effects were evaluated in 15 × 10^3^ cells, while in our study the number of cells used was 40 × 10^3^, reflecting the important inhibition capability of the compounds tested herein. Interestingly, in this work, it was shown that chloride naphthoquinone amino acid derivatives exhibited a very similar effect to Taxol, a drug that is commonly used in cervical cancer cell treatment [49]. Remarkably, the chloride naphthoquinone amino acid derivatives almost doubled the effect of Taxol in SiHa and C33-A cells. Cancer drugs are generally aimed at reducing tumor cell proliferation; however, it is desirable to inhibit other cancer-prone cell processes like migration and invasion are desirable as well. In this regard, it has been shown that natural compound juglone can reduce cell invasion via matrix metalloproteinase in ovarian cancer cells [50]. In pancreatic cancer cells, juglone inhibits invasion at an IC50 of 21 µM [51]. Additionally, Zhao et al. showed hampering of the migration and invasion process in HeLa and C-33A cells using 10 to 100 µM of the similar compound [52], which showed a concentration two to five times higher than the NQ amino acids concentration tested by our group.

Interestingly, in our study, HaCaT cell migration inhibition was higher than in SiHa, CaLo, and C33-A cells, while in the invasion assay, the tumorigenic SiHa, CaLo, and C33-A cells were more sensitive. Interestingly, the FNQCl treatment in HaCaT cells showed the strongest effect. Three NQ-amino acids (NNQCl, ANQCl and FNQCl) inhibited cell migration in SiHa, CaLo, C-33A and HaCaT cells independently of HPV and tumorigenic status. On the other hand, MNQCl inhibited migration of C-33A, CaLo and HaCaT while NNQ exerted a migratory effect in SiHa, CaLo and HaCaT cells. In contrast, GNQCl uniquely inhibited migration in HPV-negative cells, as well as in C-33A and HaCaT cells. However, it should be noted that this observation is independent of HPV status because this compound inhibited invasion in SiHa and CaLo cells which hold HPV16 and 18, respectively, as well as in HPV-negative C-33A cells. The differences in the response to NQ-amino acids can be a response to the genes regulated by each compound. Cordova-Rivas et al. showed MMP9 downregulation resulting from the overexpression of miR-34a-5p and miR-34c-5p, but not miR-34b-5p, notwithstanding the sequence homology of 83% between the 5p strands of miR-34a, b and c and presenting 3’UTR miRNA binding sites of MMP9 [14]. Additionally, it should be noted that miR-34a-5p and miR-34b-5p and miR-34c-5p have a six-nucleotide difference while miR-34b-5p and miR-34c-5p have only two different nucleotides [15]. Therefore, similar gene regulation was expected between miR-34b-5p and miR-34c-5p; however, the modulation occurred between miR-34a-5p and miR-34c-5p. In a similar manner, MMP2 was uniquely downregulated by miR-34a-3p overexpression even though miR-34b-3p and miR-34c-3p were overexpressed [14], showing gene regulation complexity dictated by cell specificity. The invasion effect in the cells tested was less uniform than that observed in cell migration in response to NQ-amino acid compounds. ANQCl and GNQCl exerted a significant invasion reduction in SiHa, C-33A and CaLo, with a null effect in HaCaT cells, while MNQCl only affected C-33A, CaLo and HaCaT cells. Hypothetically, ANQCl and GNQCl can be used to inhibit cell invasion, since only the tumorigenic cell lines were affected; nevertheless, it should be noted that additional experiments are needed to address its complete mechanism. Cell migration is associated with several specific actin filament-based structures, including lamellipodia, filopodia, invadopodia and blebs, and with cell–cell adhesion, with cell–extracellular matrix adhesion sharing several mechanisms with invasion [53]. However, cell invasion is characterized by degradation of matrix barriers and disruption of cell–cell and cell–matrix adhesion, among other characteristics [54]. The discrepancy between migration and invasion could be accounted for by the types of proteins targeted by NQ-amino acids like metalloproteins, AXL, WNK1 [55], integrin β1, Shp2 and Src kinase [56], among others. Our research group is working on omics approaches to characterize gene activation and/or inactivation in response to NQ amino acids. Numerous proteins participate in cell proliferation, migration and invasion homeostasis. It has been reported that p53 is mutated in 50% of all carcinomas [57] and its effectors are dysregulated via oncoprotein E6 in cervical cancer cells [13]. Therefore, p53 expression recuperation or overexpression are desirable in the fight against cervical cancer. Interestingly, it has been reported that NQs can lead to an increase in p53 protein levels in pancreatic cancer cells [57] and melanoma [58], colorectal cancer [59] and cervical cancer cells [60]. Additionally, it is well known that SNAIL, ZEB1, STAT3 and Myc are involved in stem cell migration and invasion processes. Interestingly, the mRNAs of SNAIL, ZEB1, STAT3 and Myc are targeted by miR-34a-5p, an effector of p53 [16,61]. In the present work, p53 expression was increased in SiHa and CaLo cells treated with NQ-amino acids; the expression was comparable to that reported in Munagala et al.’s work on HPV-positive cell lines. Nevertheless, it should be noted that in Mungala et al.’s work, no effect was recorded in C-33A cells with Tanshinone IIA (Tan II) [60], while in our work ANQCl and FNQCl provoked the induction of p53 expression. Interestingly, in HaCaT cells, increased p53 expression was only found to be associated with FNQCl exposure. The differing sensitivity to NQ-amino acids between cell lines can be attributed to several factors like the reparation system, the grade of tumorigenesis, the type and number of HPV, and the basal activity of p53 [62]. Generally, increased P53 expression is accompanied by miR-34a-5p induction; however, in this study, treatment with distinct NQ-amino acids was associated with downregulation of miR-34a-5p expression for several of the compounds in SiHa and HaCaT cells. This discrepancy could be related to miR-34a-5p promotor methylation or to the overexpression of repressors like SNAIL, ZEB1, STAT3 and Myc [61]. The treatment of SiHa and HaCaT cells with MNQCl, NNQCl ANQCl and FQNCl and with GNQCl, respectively, is conducive to a decrease in miR-34a-5p; while in the former, a decrease was observed, in the last one an increase was noted. Similarly, MNQCl and NNQCl induced a p53-independent miR-34a-5p expression decrease in SiHa cells. In contrast, in CaLo and HaCaT cells, the presence of ANQCl and FNQCl and MNQCl, respectively, was conducive to an increase in miR-34a-5p expression. In addition to p53, other miR-34a-5p inducers have been described, like the proteins Tap63, Elk1, thyroid hormone 3 [63], and Tap73 [64], which may be able to regulate miRNA in CaLo and HaCaT cells. On the other hand, it should be noted that p53 effectors like epCaM, SLUG, NOTCH, miR-143, and miR-200, among others, could be expected to be present [65]. Therefore, the linked p53 and miR-34a-5p could have a partial effect on proliferation, migration and invasion. In addition, it should be stated that a p53 increase is not necessarily indicative of the functionality of the protein. However, the relationship between a p53 increase and miR-34a-5p expression has been well documented in different origin cell types, like human chondrocytes [66], endothelia [67], colon [68], and renal cancer cells [69], among others. In C-33A, a mutation has been reported in codon 273 of the DNA-binding domains of p53 protein; this is conducive to a change in arginine being caused by cysteine [40]. Additionally, Wang et al. reported a null expression of miR-34a-5p in C-33A cells [13].

In our work, increased p53 expression was observed in C-33A treated with NQ-amino acids, whereas no miR-34a-5p expression was detected. However, p53 has a plethora of effectors, like epCaM, SLUG, NOTCH, miR-143, miR-200 among others, and its expression can regulate different cellular process [65]. A positive relationship between p53 and miR-34 has been reported [11,13,19]. However, in our work, we found this association in CaLo and HaCaT cells, while in SiHa cells the opposite association was recorded. Interestingly, in our research group, we observed a decrease in miR-34a-5p expression accompanying an increase in p53 protein expression in SiHa cells in response to UVB and NQ-amino acids. Therefore, a special type of regulation may be occurring in SiHa cells, and this requires further study. Remarkably, increased p53 expression was correlated with proliferation, migration and invasion inhibition in response to NQ-amino acids exposure. Therefore, a search for an in silico interaction between p53 and NQ-amino acids was carried out. Our results show that the FNQCl and NNQCI ligands interact specifically and favorably with the p53 protein in its active region, with a particularly notable interaction between the FNQCI ligand and Cys B:242 occurring via a pi–sulfur interaction with the aromatic ring of the ligand. This finding is especially relevant, as Cys242 is documented as an essential amino acid that helps to stabilize the DNA-binding domain of p53 due to its role in coordinating the zinc cofactor, as described in previous studies [70]. The observed pi–sulfur interaction between Cys B:242 and the aromatic portion of FNQCI represents the primary interaction within this ligand–receptor complex, suggesting a high binding stability that aligns with the interaction energy values previously described for these types of bonds [71]. As noted, sulfur-containing amino acids, particularly cysteine, are highly polarizable, allowing them to establish strong, directional van der Waals interactions with aromatic systems. This confers a thermodynamic advantage that can be crucial for structural stabilization in membrane proteins and intracellular proteins like p53 [70]. Furthermore, the interaction between FNQCI and Met A:243 through a second pi–sulfur bond further strengthens the stability of the complex. This may indicate a cooperative mechanism whereby both pi–sulfur interactions contribute to stabilizing p53’s active conformation, favoring its DNA-binding ability and thereby enhancing its function as a tumor suppressor. However, additional biochemical and functional studies are required to determine whether these interactions influence p53 stability or transcriptional activity. The interactions between cysteine and aromatic systems can significantly contribute to protein stability in systems where flexibility and structural adaptation are essential for activity [72]. On the other hand, the NNQCI ligand showed important interactions via hydrogen bonds with Ser A:241 and Arg B:248, which have also been described as recurring binding sites in p53. These interactions may be relevant for the orientation and positioning of the ligand within the protein’s active region, complementing FNQCI’s stabilizing contribution. However, it is the specific interaction with Cys B:242 that appears to play a decisive role in the overall stability of the complex, consistent with previous reports highlighting the importance of interactions with sulfur-containing amino acids for p53 functionality [70].

## 4. Materials and Methods

### 4.1. ABTS Inhibition Assay

The antioxidant capacity of the synthesized naphthoquinones was assessed by determining the compounds’ capacity to scavenge the free radical 2.2-azinobis-3-ethylbenzothiazoline-6-sulphonic acid (ABTS) according to the method described by Managa et al. [73], with some modifications. The ABTS radical 7mM was prepared in a phosphate solution and potassium persulfate 2.45 mM. Afterwards, the solution was stored in darkness for 16 h at 25 °C and diluted with the phosphate solution until an absorbance of 0.70 ± 0.03 was achieved at 734 nm. Each NQ was dissolved with DMSO to obtain its corresponding IC50 and the final volume was made up to 100 µL with distilled water; 1 mL of the radical solution was added. The absorbance of NQ and a control (100 µL of phosphate buffer instead of NQ) was measured at 734 nm. The results were expressed as % inhibition of the radical, calculated as follows:

% inhibition = [(Abs Control − Abs sample)/(Abs Control)] × 100

Additionally, for each naphthoquinone, a blank was considered to subtract the potential antioxidant capacity of DMSO. The antioxidant capacity of synthesized naphthoquinones was compared to Trolox 0.2%.

### 4.2. Reducing Power Assay

The reducing power of the synthesized naphthoquinones was measured as described by Ribeiro et al. [74], with modifications. The reaction mixture contained 200 µL of the DMSO-dissolved naphthoquinone and 1 mL potassium ferricyanide (1%, *w*/*v*, in water). The mixture was incubated at 50 °C for 30 min. An aliquot of 0.5 mL of the mixture was mixed with 0.5 mL of distilled water and 0.23 mL of ferric chloride (0.1%, *w*/*v*, in water) and the absorbance was measured at 700 nm against blanks that contained all reagents except the naphthoquinone. Additionally, for each naphthoquinone, a blank was considered to subtract the potential antioxidant capacity of DMSO. The reducing power of synthesized naphthoquinones was compared to Trolox 0.2%.

### 4.3. Culture of Cell Lines

The SiHa (tumorigenic HPV 16-positive), C-33A (tumorigenic HPV-negative), CaLo (tumorigenic HPV 18-positive) and HaCaT (immortal HPV-negative) cell lines were cultured in DMEM (Dulbecco’s Modified Eagle’s Medium (Invitrogen Corporation, Carlsbad, CA, USA)) enriched with 6% fetal bovine serum (FBS), at 37 °C in a 5% CO_2_ environment. The cell lines were donated by the laboratory of Dr. Patricio Gariglio Vidal of the Center for Research and Advanced Studies of the IPN (CINVESTAV-IPN).

### 4.4. Treatment of Cells with Naphthoquinone-Amino Acid

A total of 200,000 cells were seeded in p60 boxes. Subsequently, 3 mL of DMEM with 6% SFB was added and exposed to 625 to 200 mM of naphthoquinone-amino acids for cells proliferation analysis. For migration, invasion, Western blotting and RT-PCR evaluation, the IC50 of NNQ, ANQCl, MNQCl, GNQCl, NNQCl and FNQCl was used. Cells exposed to DMSO were used as a solvent for the NQ compounds and were used as a control. Treated cells were shaken and incubated at 37 °C for 24 h for migration and invasion assays and 48 h for Western blotting and RT-qPCR.

### 4.5. Cell Proliferation Assay

Firstly, 20,000 treated cells were seeded per well, incubated for 72 h, and then washed with 1X PBS. The cells were fixed with 100% methanol cold, stained with 0.1% crystal violet for 20 min, and washed three times with distilled water. Finally, 10% acetic acid was added and the absorbance was read at 600 nm on a spectrophotometer (Multiskan GO spectrophotometer, Thermo Scientific™, Waltham, MA, USA).

### 4.6. Determination of IC50

GraphPad Prism8 software was used to determine IC50 from the percentage of cell inhibition obtained in three independent cell proliferation curve assays.

### 4.7. Western Blotting

Cells were lysed using RIPA lysis buffer (150 mM NaCl, 50 mM Tris-HCl pH 8, 0.1% SDS, 1% NP40, 0.5 mM AEBSF, 10 mM Deoxycholate and protease inhibitors: E-64, Aprotinin, Bestatin, Leupeptin and Pepstatin). The resulting proteins were quantified by Bradford’s method.

Next, 60 μg of protein was prepared in 1X Laemmli buffer (5X Laemmli (250 mM tris-HCl pH 6.8. 10% SDS, 50% glycerol, 10% beta-mercapto-ethanol, and 0.25% bromophenol blue) then boiled for 5 min. These samples were loaded onto a 10% SDS-polyacrylamide gel and subjected to electrophoresis. The proteins were transferred to a low-fluorescence PVDF membrane (BioRad supported PVDF membrane 0.22 µm) at 300 mA for 150 min. The membrane was blocked with a PBS-tween 1X-milk 5% for 2 h. Subsequently, it was incubated overnight with primary antibodies p53 (sc-126) and β-actin (sc-58673) diluted at 1:1000 and 1:3000 ratios, respectively. Anti-mouse secondary antibody was diluted 1:30,000 and incubated for one hour at room temperature. Finally, protein detection was performed by chemiluminescence using the ECL kit (Santa Cruz Biotechnology, Dallas, TX, USA), and the results were captured and analyzed on the Bio-Rad ChemiDoc MP imaging System.

### 4.8. Small RNA Extraction

Small RNA isolation was performed using a “mirVanaTM miRNA Isolation Kit” (AM1560) following the manufacturer’s specifications. Briefly, the medium was collected from cells treated with NQ-amino acid compounds, centrifuged to recover floating cells, and lysis solution was added to both the culture box and cell pellet. The lysate was mixed with “miRNA Homogenate Additive” solution, incubated on ice, chloroform was added, and the mixture was centrifuged. The upper phase was separated, 100% ethanol was added and the lysate/ethanol mixture was filtered to isolate small RNA. RNA was eluted with nuclease-free water at 95 °C. To verify the presence and integrity of small RNA, a 2% agarose gel was utilized. Finally, the amount and purity of RNA were evaluated using the relative absorbance ratio at 260/280 in a spectrophotometer (Thermo Scientific Multiskan Go, Waltham, MA, USA).

### 4.9. RT-qPCR

cDNA synthesis of each miRNA was achieved using specific primers and quantified by 2^−ΔΔCt^. RT reactions were performed with 10 ng of small RNAs, 12.5 nM RT primer, RT buffer (1X), 2 mM dNTPs mix and 40 U of M-MVL reverse transcriptase (Invitrogen, Carlsbad, CA, USA, cat: 28025013) in a volume of 10 μL. The reaction cycle was performed for 60 min at 37 °C followed by 1 cycle lasting 15 min at 70 °C. qPCR reactions were performed using total volumes of 10 μL containing 1/3 of the final RT volume, Eva Green Dye (0.6×) (Biotium, Fremont, CA, USA, cat: 31000-T), 0.5 μM forward primer, 0.3 μM reverse primer, 200 nM dNTPs and 1 U of Taq polymerase (Invitrogen, cat: 11615-010). The cycle amplification reactions were as follows: 95 °C for 10 min, 40 cycles at 95 °C for 5 s, and 74 °C for 1 min using the CFX Opus 96 real-time PCR instrument (Bio-Rad Life Science, Hercules, CA, USA).

### 4.10. Cell Migration Assay

After NQ-amino acids were exposed to IC50, cells were treated with 1800 ng of mitomycin C for two hours. Subsequently, 80,000 cells per well were seeded on a polyester insert with a diameter pore of 8 μm in 2% DMEM. This insert was placed inside 10 mm diameter well with 600 μL of 10% DMEM. The cells were incubated at 37 °C in 5% CO_2_ for 72 h. After incubation, the medium was removed and excess non-migrated cells were removed from the top of the membrane using absorbent cotton. The migrated cells were fixed with 100% methanol 4 °C, washed with 1X PBS, and stained with 0.1% crystal violet. Finally, cells were washed three times with distilled water and membranes were released from the insert, and incubated in 10% acetic acid for 1 h. The absorbance of 100 μL of the solution was measured at 600 nm.

### 4.11. Cell Invasion Assay

Matrigel (Corning^®^ Matrigel^®^, Matrix REF: 356237, Corning, NY, USA) was added at a concentration of 0.5 mg/mL on a polyester membrane with a pore diameter of 8 μm and processed in the same manner as in the cell migration assay.

### 4.12. Molecular Docking Studies

In order to investigate the interaction between NQ-derived compounds and the p53 protein, modeling-based molecular docking studies were performed. To optimize the structure of the compounds, a minimum energy calculation was performed using the molecular mechanics method MMFF in Spartan 14 [75]. Subsequently, the protonation state was determined using the algorithm implemented in the Open Babel software. The ligands were prepared in AutodockTools [76]; Gasteiger-type charges were added and the rotational bonds were defined. The coordinates of the PDB code 2AC0 [42] were used for the receptor. The receptor structure was subjected to an exhaustive review using the DockPrep algorithm of the Chimera program [77], avoiding structural errors or missing amino acids. Subsequently, an energy minimization was performed in the same way using the Chimera module. In the same way as with the ligands, Kollman-type electrical charges were added to the receptor. Finally, ligand and receptor preparation were analyzed in AutodockTools using the following features: molecular docking was performed under a blind and rigid approach, with the following coordinates: x = 28.830, y = −1.308, z = 10.812, and a mesh calculation area of 126 × 126 × 126 points, with a spacing of 0.37 Å.

The conformational search method was analyzed using genetic algorithms [78], with an initial population size of 150 and a maximum number of 100 runs, run-termination criterion of a maximum of 27,000,000 generations or a maximum of 250,000 energy evaluations. A crossover rate of 0.8 and a gene mutation rate of 0.02 were used, and finally, the 10 best conformations of the ligands were selected based on the minimum energy value.

Visualization and analysis of the nonbonded interactions as hydrogen bonds of the best poses were achieved using Discovery Studio Visualizer v21.1.0.20298 software [79].

### 4.13. Statistics

The data obtained were subjected to the Shapiro–Wilk test to determine normal distribution. Subsequently, a one-way analysis of variance (ANOVA) was performed to determine the statistically significant difference and a post hoc multiple-comparison test (Dunnett) was applied.

## 5. Conclusions

Differently composed NQs were found to be capable of exerting differential responses in a cell line-dependent manner, confirming the difficulty of treating particular background cancer cells with standard therapy and highlighting the importance of developing novel compounds. However, interestingly, the chloride NQ-amino acids were more effective at hampering proliferation of tumorigenic cell lines than of immortalized cells, suggesting common susceptibility to this kind of compound and indicating that it has potential for use in a more advanced tumor phase. Interestingly, the compounds exerted important inhibition of migration and invasion, highlighting the scope of action of the compounds on different tumorigenic abilities. Additionally, differently structured compounds also had cell line-specific effects on p53 induction. NNQCl, ANQCl, and FNQCl induced p53 expression in SiHa cells while, NNQ, GNQCl, ANQCl, and FNQCl provoked p53 overexpression in C-33A cells. In CaLo cells, GNQCl, ANQCl, FNQCl treatment resulted in an increase in p53, whilst in HaCaT, only the FNQCl compound triggered p53 expression. Surprisingly, NNQ, NNQCl, MNQCl and GNQCl inhibited proliferation, migration and invasion in a p53-miR-34a-5p-independent way. Therefore, different miR-34a-5p modulators should be explored as potential therapeutic targets. Molecular docking suggests that there are favorable interactions between FNQCl and NNQCl with residues located within the DNA-binding domain of p53, including Cys242. These computational findings should be supported by future studies to elucidate the molecular mechanisms of the biological effects observed in vitro.

## Figures and Tables

**Table 1:** Determination of the IC_50_ with NQ-amino acids in cervical cancer cells.

COMPOUND	SiHa IC_50_ (μM)	C-33A IC_50_ (μM)	CaLo IC_50_ (μM)	HaCaT IC_50_ (μM)
ANQ	31.76	17.010	33.400	46.400
MNQ	19.34	14.060	29.230	30.600
GNQ	7.721	6.480	9.222	11.910
FNQ	25.47	20.820	41.680	69.24
NNQ	3.752	5.468	7.370	10.370
NNQCl	5.318	4.837	6.035	6.986
MNQCl	7.274	3.555	5.247	7.882
GNQCl	9.333	3.287	5.121	5.302
ANQCl	6.604	3.459	5.613	8.018
FNQCl	10.700	3.787	4.991	6.752

**Table 2:** Receptor–ligand interactions between p53 with NQCI ligands.

Ligand	ΔG (kcal/mol)	ki (µmol)	Interaction Type	Receptor Residue
NNQ	−7.75	2.10	Hydrogen bond	Arg B:248
				Ser A:241
				Guanine E:12
ANQCI	−7.84	1.8	Hydrogen bond	Adenine E:6
				Arg B:248
				Guanine E:8
			Pi–alkyl	Guanine F:8
NNQCI	−8.05	1.27	Hydrogen bond	Ser A:241
				Arg B:248
				Guanine E:8
				Guanine E:12
FNQCI	−8.4	0.70	Hydrogen bond	Met A:243
				Guanine E:12
				Cys B:242
			Pi–sulphur	Met A:243
GNQCI	−7.64	2.52	Hydrogen bond	Arg B:248
				Guanine E:8
				Guanine E:12
MNQCI	−7.67	2.39	Hydrogen bond	Arg B:248
				Guanine E:12
			Alkyl	Met A:243

## Data Availability

The original contributions presented in this study are included in the article/Supplementary Materials. Further inquiries can be directed to the corresponding author.

## Supplementary Materials

The following supporting information can be downloaded at: https://www.mdpi.com/article/10.3390/ijms27135703/s1.

## References

[1] Sung H., Ferlay J., Siegel R.L., Laversanne M., Soerjomataram I., Jemal A., Bray F. (2021). Global Cancer Statistics 2020: GLOBOCAN Estimates of Incidence and Mortality Worldwide for 36 Cancers in 185 Countries. CA Cancer J. Clin..

[2] Woodworth C.D., Cheng S., Simpson S., Hamacher L., Chow L.T., Broker T.R., DiPaolo J.A. (1992). Recombinant retroviruses encoding human papillomavirus type 18 E6 and E7 genes stimulate proliferation and delay differentiation of human keratinocytes early after infection. Oncogene.

[3] zur Hausen H. (1999). Immortalization of human cells and their malignant conversion by high risk human papillomavirus genotypes. Semin. Cancer Biol..

[4] Werness B.A., Levine A.J., Howley P.M. (1990). Association of human papillomavirus types 16 and 18 E6 proteins with p53. Science.

[5] Hubbert N.L., Sedman S.A., Schiller J.T. (1992). Human papillomavirus type 16 E6 increases the degradation rate of p53 in human keratinocytes. J. Virol..

[6] Dyson N., Howley P.M., Munger K., Harlow E. (1989). The human papilloma virus-16 E7 oncoprotein is able to bind to the retinoblastoma gene product. Science.

[7] Duensing S., Duensing A., Flores E.R., Do A., Lambert P.F., Munger K. (2001). Centrosome abnormalities and genomic instability by episomal expression of human papillomavirus type 16 in raft cultures of human keratinocytes. J. Virol..

[8] Munger K., Basile J.R., Duensing S., Eichten A., Gonzalez S.L., Grace M., Zacny V.L. (2001). Biological activities and molecular targets of the human papillomavirus E7 oncoprotein. Oncogene.

[9] Ji Q., Hao X., Zhang M., Tang W., Yang M., Li L., Xiang D., Desano J.T., Bommer G.T., Fan D. (2009). MicroRNA miR-34 inhibits human pancreatic cancer tumor-initiating cells. PLoS ONE.

[10] Corney D.C., Flesken-Nikitin A., Godwin A.K., Wang W., Nikitin A.Y. (2007). MicroRNA-34b and MicroRNA-34c are targets of p53 and cooperate in control of cell proliferation and adhesion-independent growth. Cancer Res..

[11] Bommer G.T., Gerin I., Feng Y., Kaczorowski A.J., Kuick R., Love R.E., Zhai Y., Giordano T.J., Qin Z.S., Moore B.B. (2007). p53-mediated activation of miRNA34 candidate tumor-suppressor genes. Curr. Biol..

[12] Kumamoto K., Spillare E.A., Fujita K., Horikawa I., Yamashita T., Appella E., Nagashima M., Takenoshita S., Yokota J., Harris C.C. (2008). Nutlin-3a activates p53 to both down-regulate inhibitor of growth 2 and up-regulate mir-34a, mir-34b, and mir-34c expression, and induce senescence. Cancer Res..

[13] Wang X., Wang H.K., McCoy J.P., Banerjee N.S., Rader J.S., Broker T.R., Meyers C., Chow L.T., Zheng Z.M. (2009). Oncogenic HPV infection interrupts the expression of tumor-suppressive miR-34a through viral oncoprotein E6. RNA.

[14] Cordova-Rivas S., Fraire-Soto I., Mercado-Casas Torres A., Servin-Gonzalez L.S., Granados-Lopez A.J., Lopez-Hernandez Y., Reyes-Estrada C.A., Gutierrez-Hernandez R., Castaneda-Delgado J.E., Ramirez-Hernandez L. (2019). 5p and 3p Strands of miR-34 Family Members Have Differential Effects in Cell Proliferation, Migration, and Invasion in Cervical Cancer Cells. Int. J. Mol. Sci..

[15] Granados-Lopez A.J., Ruiz-Carrillo J.L., Servin-Gonzalez L.S., Martinez-Rodriguez J.L., Reyes-Estrada C.A., Gutierrez-Hernandez R., Lopez J.A. (2017). Use of Mature miRNA Strand Selection in miRNAs Families in Cervical Cancer Development. Int. J. Mol. Sci..

[16] Pan W., Chai B., Li L., Lu Z., Ma Z. (2023). p53/MicroRNA-34 axis in cancer and beyond. Heliyon.

[17] Tanaka N., Toyooka S., Soh J., Kubo T., Yamamoto H., Maki Y., Muraoka T., Shien K., Furukawa M., Ueno T. (2012). Frequent methylation and oncogenic role of microRNA-34b/c in small-cell lung cancer. Lung Cancer.

[18] Hagman Z., Larne O., Edsjo A., Bjartell A., Ehrnstrom R.A., Ulmert D., Lilja H., Ceder Y. (2010). miR-34c is downregulated in prostate cancer and exerts tumor suppressive functions. Int. J. Cancer.

[19] He L., He X., Lim L.P., de Stanchina E., Xuan Z., Liang Y., Xue W., Zender L., Magnus J., Ridzon D. (2007). A microRNA component of the p53 tumour suppressor network. Nature.

[20] Cai K.M., Bao X.L., Kong X.H., Jinag W., Mao M.R., Chu J.S., Huang Y.J., Zhao X.J. (2010). Hsa-miR-34c suppresses growth and invasion of human laryngeal carcinoma cells via targeting c-Met. Int. J. Mol. Med..

[21] Sikka H.C., Shimabukuro R.H., Zweig G. (1972). Studies on Effect of Certain Quinones: I. Electron Transport, Photophosphorylation, and CO(2) Fixation in Isolated Chloroplasts. Plant Physiol..

[22] Kosiha A., Parthiban C., Elango K.P. (2017). Synthesis, characterization and DNA binding/cleavage, protein binding and cytotoxicity studies of Co(II), Ni(II), Cu(II) and Zn(II) complexes of aminonaphthoquinone. J. Photochem. Photobiol. B.

[23] Li C.J., Li Y.Z., Pinto A.V., Pardee A.B. (1999). Potent inhibition of tumor survival in vivo by beta-lapachone plus taxol: Combining drugs imposes different artificial checkpoints. Proc. Natl. Acad. Sci. USA.

[24] Chen J., Huang Y.W., Liu G., Afrasiabi Z., Sinn E., Padhye S., Ma Y. (2004). The cytotoxicity and mechanisms of 1,2-naphthoquinone thiosemicarbazone and its metal derivatives against MCF-7 human breast cancer cells. Toxicol. Appl. Pharmacol..

[25] Kongkathip N., Kongkathip B., Siripong P., Sangma C., Luangkamin S., Niyomdecha M., Pattanapa S., Piyaviriyagul S., Kongsaeree P. (2003). Potent antitumor activity of synthetic 1,2-Naphthoquinones and 1,4-Naphthoquinones. Bioorg. Med. Chem..

[26] Kongkathip N., Luangkamin S., Kongkathip B., Sangma C., Grigg R., Kongsaeree P., Prabpai S., Pradidphol N., Piyaviriyagul S., Siripong P. (2004). Synthesis of novel rhinacanthins and related anticancer naphthoquinone esters. J. Med. Chem..

[27] Pedersen J.A. (2002). On the application of electron paramagnetic resonance in the study of naturally occurring quinones and quinols. Spectrochim. Acta A Mol. Biomol. Spectrosc..

[28] Coates C.S., Ziegler J., Manz K., Good J., Kang B., Milikisiyants S., Chatterjee R., Hao S., Golbeck J.H., Lakshmi K.V. (2013). The structure and function of quinones in biological solar energy transduction: A cyclic voltammetry, EPR, and hyperfine sub-level correlation (HYSCORE) spectroscopy study of model naphthoquinones. J. Phys. Chem. B.

[29] Smithson C.S., MacDonald D.J., Matt Letvenuk T., Carello C.E., Jennings M., Lough A.J., Britten J., Decken A., Preuss K.E. (2016). A 1,2,3-dithiazolyl-o-naphthoquinone: A neutral radical with isolable cation and anion oxidation states. Dalton Trans..

[30] Tarabek J., Wen J., Dron P.I., Pospisil L., Michl J. (2018). EPR Spectroscopy of Radical Ions of a 2,3-Diamino-1,4-naphthoquinone Derivative. J. Org. Chem..

[31] Wardman P. (2001). Electron transfer and oxidative stress as key factors in the design of drugs selectively active in hypoxia. Curr. Med. Chem..

[32] Ham S.W., Choe J.I., Wang M.F., Peyregne V., Carr B.I. (2004). Fluorinated quinoid inhibitor: Possible “pure” arylator predicted by the simple theoretical calculation. Bioorg. Med. Chem. Lett..

[33] Kar S., Wang M., Ham S.W., Carr B.I. (2006). Fluorinated Cpd 5, a pure arylating K-vitamin derivative, inhibits human hepatoma cell growth by inhibiting Cdc25 and activating MAPK. Biochem. Pharmacol..

[34] Park H., Carr B.I., Li M., Ham S.W. (2007). Fluorinated NSC as a Cdc25 inhibitor. Bioorg. Med. Chem. Lett..

[35] Subramaniya B.R., Srinivasan G., Sadullah S.S., Davis N., Subhadara L.B., Halagowder D., Sivasitambaram N.D. (2011). Apoptosis inducing effect of plumbagin on colonic cancer cells depends on expression of COX-2. PLoS ONE.

[36] Rivera-Avalos E., de Loera D., Araujo-Huitrado J.G., Escalante-Garcia I.L., Munoz-Sanchez M.A., Hernandez H., Lopez J.A., Lopez L. (2019). Synthesis of Amino Acid-Naphthoquinones and In Vitro Studies on Cervical and Breast Cell Lines. Molecules.

[37] Cordova-Rivas S., Araujo-Huitrado J.G., Rivera-Avalos E., Escalante-Garcia I.L., Duron-Torres S.M., Lopez-Hernandez Y., Hernandez-Lopez H., Lopez L., de Loera D., Lopez J.A. (2020). Differential Proliferation Effect of the Newly Synthesized Valine, Tyrosine and Tryptophan-Naphthoquinones in Immortal and Tumorigenic Cervical Cell Lines. Molecules.

[38] Sahoo B.M., Banik B.K., Borah P., Jain A. (2022). Reactive Oxygen Species (ROS): Key Components in Cancer Therapies. Anticancer Agents Med. Chem..

[39] de Oliveira A.S., Palomino-Salcedo D.L., Zapp E., Brondani D., Hoppe T.D., Brondani P.B., Meier L., Johann S., Ferreira L.L.G., Andricopulo A.D. (2020). Molecular Docking and Quantum Studies of Lawsone Dimers Derivatives: New Investigation of Antioxidant Behavior and Antifungal Activity. Curr. Top. Med. Chem..

[40] Scheffner M., Munger K., Byrne J.C., Howley P.M. (1991). The state of the p53 and retinoblastoma genes in human cervical carcinoma cell lines. Proc. Natl. Acad. Sci. USA.

[41] Watson J. (2013). Oxidants, antioxidants and the current incurability of metastatic cancers. Open Biol..

[42] Zahra K., Patel S., Dey T., Pandey U., Mishra S.P. (2021). A study of oxidative stress in cervical cancer- an institutional study. Biochem. Biophys. Rep..

[43] Sayin V.I., Ibrahim M.X., Larsson E., Nilsson J.A., Lindahl P., Bergo M.O. (2014). Antioxidants accelerate lung cancer progression in mice. Sci. Transl. Med..

[44] Beretta G.L., Ribaudo G., Menegazzo I., Supino R., Capranico G., Zunino F., Zagotto G. (2017). Synthesis and Evaluation of New Naphthalene and Naphthoquinone Derivatives as Anticancer Agents. Arch. Pharm..

[45] Meem M.H., Yusuf S.B., Al Abbad S.S., Rahman S., Al-Gawati M., Albrithen H., Alodhayb A.N., Uddin K.M. (2024). Exploring the anticancer and antibacterial potential of naphthoquinone derivatives: A comprehensive computational investigation. Front. Chem..

[46] Estolano-Cobián A., Noriega-Iribe E., Díaz-Rubio L., Padrón J.M., Brito-Perea M., Cornejo-Bravo J.M., Chávez D., Rivera R.R., Quintana-Melgoza J.M., Cruz-Reyes J. (2020). Antioxidant, antiproliferative, and acetylcholinesterase inhibition activity of amino alcohol derivatives from 1,4-naphthoquinone. Med. Chem. Res..

[47] de Moraes T.A., Filha M.J., Camara C.A., Silva T.M., Soares B.M., Bomfim I.S., Pessoa C., Ximenes G.C., Silva Junior V.A. (2014). Synthesis and cytotoxic evaluation of a series of 2-amino-naphthoquinones against human cancer cells. Molecules.

[48] Marastoni M., Trapella C., Scotti A., Fantinati A., Ferretti V., Marzola E., Eleonora G., Gavioli R., Preti D. (2017). Naphthoquinone amino acid derivatives, synthesis and biological activity as proteasome inhibitors. J. Enzym. Inhib. Med. Chem..

[49] Wang J. (2020). Combination Treatment of Cervical Cancer Using Folate-Decorated, pH-Sensitive, Carboplatin and Paclitaxel Co-Loaded Lipid-Polymer Hybrid Nanoparticles. Drug Des. Dev. Ther..

[50] Fang F., Qin Y., Qi L., Fang Q., Zhao L., Chen S., Li Q., Zhang D., Wang L. (2015). Juglone exerts antitumor effect in ovarian cancer cells. Iran. J. Basic Med. Sci..

[51] Avci E., Arikoglu H., Erkoc Kaya D. (2016). Investigation of juglone effects on metastasis and angiogenesis in pancreatic cancer cells. Gene.

[52] Zhao X., Zhu W., Zhang R., Zhang M., Zhao J., Hou J., Zhang W. (2019). Targeted juglone blocks the invasion and metastasis of HPV-positive cervical cancer cells. J. Pharmacol. Sci..

[53] Guan X., Guan X., Dong C., Jiao Z. (2020). Rho GTPases and related signaling complexes in cell migration and invasion. Exp. Cell Res..

[54] Perlikos F., Harrington K.J., Syrigos K.N. (2013). Key molecular mechanisms in lung cancer invasion and metastasis: A comprehensive review. Crit. Rev. Oncol. Hematol..

[55] Jaykumar A.B., Jung J.U., Parida P.K., Dang T.T., Wichaidit C., Kannangara A.R., Earnest S., Goldsmith E.J., Pearson G.W., Malladi S. (2021). WNK1 Enhances Migration and Invasion in Breast Cancer Models. Mol. Cancer Ther..

[56] Conway J.R.W., Joshi O., Kaivola J., Follain G., Gounis M., Kuhl D., Ivaska J. (2025). Dynamic regulation of integrin beta1 phosphorylation supports invasion of breast cancer cells. Nat. Cell Biol..

[57] Lee S.H., Shen G.N., Jung Y.S., Lee S.J., Chung J.Y., Kim H.S., Xu Y., Choi Y., Lee J.W., Ha N.C. (2010). Antitumor effect of novel small chemical inhibitors of Snail-p53 binding in K-Ras-mutated cancer cells. Oncogene.

[58] Li Z., Liu X., Li M., Chai J., He S., Wu J., Xu J. (2020). Juglone potentiates BRAF inhibitor-induced apoptosis in melanoma through reactive oxygen species and the p38–p53 pathway. Mol. Med. Rep..

[59] Raghu D., Karunagaran D. (2014). Plumbagin downregulates Wnt signaling independent of p53 in human colorectal cancer cells. J. Nat. Prod..

[60] Munagala R., Aqil F., Jeyabalan J., Gupta R.C. (2015). Tanshinone IIA inhibits viral oncogene expression leading to apoptosis and inhibition of cervical cancer. Cancer Lett..

[61] Li W.J., Wang Y., Liu R., Kasinski A.L., Shen H., Slack F.J., Tang D.G. (2021). MicroRNA-34a: Potent Tumor Suppressor, Cancer Stem Cell Inhibitor, and Potential Anticancer Therapeutic. Front. Cell Dev. Biol..

[62] Hietanen S., Lain S., Krausz E., Blattner C., Lane D.P. (2000). Activation of p53 in cervical carcinoma cells by small molecules. Proc. Natl. Acad. Sci. USA.

[63] Slabakova E., Culig Z., Remsik J., Soucek K. (2017). Alternative mechanisms of miR-34a regulation in cancer. Cell Death Dis..

[64] Agostini M., Tucci P., Killick R., Candi E., Sayan B.S., Rivetti di Val Cervo P., Nicotera P., McKeon F., Knight R.A., Mak T.W. (2011). Neuronal differentiation by TAp73 is mediated by microRNA-34a regulation of synaptic protein targets. Proc. Natl. Acad. Sci. USA.

[65] Muller P.A., Vousden K.H., Norman J.C. (2011). p53 and its mutants in tumor cell migration and invasion. J. Cell Biol..

[66] Zhou M., Liu B., Ye H.M., Hou J.N., Huang Y.C., Zhang P., Gao L., Qin H.T., Yang Y.F., Zeng H. (2024). ROS-induced imbalance of the miR-34a-5p/SIRT1/p53 axis triggers chronic chondrocyte injury and inflammation. Heliyon.

[67] Manakanatas C., Ghadge S.K., Agic A., Sarigol F., Fichtinger P., Fischer I., Foisner R., Osmanagic-Myers S. (2022). Endothelial and systemic upregulation of miR-34a-5p fine-tunes senescence in progeria. Aging.

[68] Lee D.H., Seok H.J., Choi J.Y., Kwon J., Shin U.S., Bae I.H. (2025). Tetrabenazine-induced miR-34a-5p suppresses the tumorigenicity of radioresistant colorectal cancer by inhibiting M2 macrophage polarization. Cell Commun. Signal.

[69] Jing Z.F., Bi J.B., Li Z., Liu X., Li J., Zhu Y., Zhang X.T., Zhang Z., Li Z., Kong C.Z. (2019). Inhibition of miR-34a-5p can rescue disruption of the p53-DAPK axis to suppress progression of clear cell renal cell carcinoma. Mol. Oncol..

[70] Joerger A.C., Ang H.C., Fersht A.R. (2006). Structural basis for understanding oncogenic p53 mutations and designing rescue drugs. Proc. Natl. Acad. Sci. USA.

[71] Gomez-Tamayo J.C., Cordomi A., Olivella M., Mayol E., Fourmy D., Pardo L. (2016). Analysis of the interactions of sulfur-containing amino acids in membrane proteins. Protein Sci..

[72] Beno B.R., Yeung K.S., Bartberger M.D., Pennington L.D., Meanwell N.A. (2015). A Survey of the Role of Noncovalent Sulfur Interactions in Drug Design. J. Med. Chem..

[73] Managa M.G., Sultanbawa Y., Sivakumar D. (2020). Effects of Different Drying Methods on Untargeted Phenolic Metabolites, and Antioxidant Activity in Chinese Cabbage (*Brassica rapa* L. subsp. chinensis) and Nightshade (*Solanum retroflexum* Dun.). Molecules.

[74] Ribeiro S.M.R., Barbosa L.C.A., Queiroz J.H., Knodler M., Schieber A. (2008). Phenolic compounds and antioxidant capacity of Brazilian mango (*Mangifera indica* L.) varieties. Food Chem..

[75] SPARTAN (2016). Wavefunction, 14.

[76] Sanner M.F. (1999). Python: A programming language for software integration and development. J. Mol. Graph. Model..

[77] Pettersen E.F., Goddard T.D., Huang C.C., Couch G.S., Greenblatt D.M., Meng E.C., Ferrin T.E. (2004). UCSF Chimera—A visualization system for exploratory research and analysis. J. Comput. Chem..

[78] Morris G.M., Goodsell D.S., Halliday R.S., Huey R., Hart W.E., Belew R.K., Olson A.J. (1998). Automated docking using a Lamarckian genetic algorithm and an empirical binding free energy function. J. Comput. Chem..

[79] Biovia D.S. (2021). Discovery Studio Visualizer v21.1.020298.

