# Supplementary material for: Naphthoquinone-Amino Acids Regulate Cellular Cancer Associated Processes, p53 and miR-34a-5p Expression in Immortal and Tumorigenic Cervical Cell Lines
Source: Int J Mol Sci. 2026 Jun 24;27(13):5703. doi: 10.3390/ijms27135703 (PMC13361552; doi:10.3390/ijms27135703)

Supplementary material of molecular docking calculations:

1. Chemical structure and xyz coordinates of optimized ligands:
   The 2D molecular structures were created using ChemBioDraw Ultra 13.0, while the 3D coordinates were generated with ChemSketch. The optimization was performed using Spartan 14.0, employing Molecular Mechanics at the Merck Molecular Force Field (MMFF) level of theory.

Figure S1. Chemical structure of **NNQ** and its optimized coordinates.

Molecular structure:

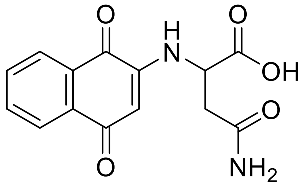

Optimized coordinates:

@<TRIPOS>MOLECULE

*****

32 33 0 0 0

SMALL

GASTEIGER

@<TRIPOS>ATOM

1 C	3.3687	-5.3073	-0.4161	C.ar	0	UNK0	-0.0610
2 C	3.3175	-6.6921	-0.3773	C.ar	0	UNK0	-0.0610
3 C	4.4904	-7.4280	-0.3502	C.ar	0	UNK0	-0.0502
4 C	5.7137	-6.7787	-0.3613	C.ar	0	UNK0	0.0326
5 C	5.7660	-5.3927	-0.4061	C.ar	0	UNK0	0.0343
6 C	4.5933	-4.6571	-0.4301	C.ar	0	UNK0	-0.0502
7 C	6.9641	-7.5531	-0.3117	C.2	0	UNK0	0.1886
8 C	8.2589	-6.8492	-0.2454	C.2	0	UNK0	0.0103
9 C	8.3265	-5.5057	-0.2929	C.2	0	UNK0	0.0792
10 C	7.0799	-4.7113	-0.4241	C.2	0	UNK0	0.2075

```
     11 O          7.1362   -3.5100   -0.5586 O.2      0  UNK0       -0.2853
     12 O          6.9303   -8.7624   -0.3072 O.2      0  UNK0       -0.2872
     13 N          9.5532   -4.8560   -0.2307 N.pl3    1  ASN1       -0.3323
     14 CA        10.8528   -5.5232    0.0000 C.3      1  ASN1        0.0684
     15 C         11.9720   -4.5606   -0.3149 C.2      1  ASN1        0.0623
     16 CB        11.0200   -6.7584   -0.8923 C.3      1  ASN1        0.0473
     17 O         13.2165   -5.0401   -0.5816 O.co2    1  ASN1       -0.5479
     18 OXT       11.7651   -3.3682   -0.3342 O.co2    1  ASN1       -0.5479
     19 CG        12.2460   -7.5253   -0.4579 C.2      1  ASN1        0.2126
     20 OD1       13.3072   -6.9541   -0.3501 O.2      1  ASN1       -0.2760
     21 ND2       12.1583   -8.8770   -0.1966 N.am     1  ASN1       -0.3293
     22 H          2.4942   -4.7596   -0.4346 H        0  UNK0        0.0618
     23 H          2.4053   -7.1747   -0.3686 H        0  UNK0        0.0618
     24 H          4.4529   -8.4589   -0.3218 H        0  UNK0        0.0625
     25 H          4.6306   -3.6262   -0.4584 H        0  UNK0        0.0625
     26 H          9.1293   -7.3972   -0.1610 H        0  UNK0        0.0676
     27 H          9.5576   -3.8786   -0.3490 H        1  ASN1        0.1468
     28 HA        10.8833   -5.8296    1.0247 H        1  ASN1        0.0564
     29 HB1       11.1330   -6.4509   -1.9109 H        1  ASN1        0.0380
     30 HB2       10.1562   -7.3837   -0.8045 H        1  ASN1        0.0380
     31 HD21      11.2941   -9.3404   -0.2848 H        1  ASN1        0.1449
     32 HD22      12.9599   -9.3783    0.0780 H        1  ASN1        0.1449
@<TRIPOS>UNITY_ATOM_ATTR
17 1
charge -1
```

Figure S2. Chemical structure of **ANQCl** and its optimized coordinates.

Molecular structure:

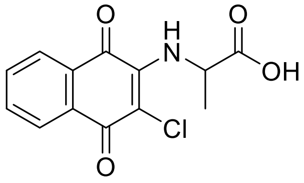

Optimized coordinates:

```
@<TRIPOS>MOLECULE
*****
 28 29 0 0 0
SMALL
GASTEIGER

@<TRIPOS>ATOM
      1 C          3.8262   -4.3378   -0.8727 C.ar     0  UNK0       -0.0610
      2 C          3.7465   -5.7179   -0.9741 C.ar     0  UNK0       -0.0610
      3 C          4.9044   -6.4770   -1.0457 C.ar     0  UNK0       -0.0502
      4 C          6.1417   -5.8541   -1.0163 C.ar     0  UNK0        0.0340
      5 C          6.2209   -4.4725   -0.9192 C.ar     0  UNK0        0.0344
      6 C          5.0636   -3.7137   -0.8455 C.ar     0  UNK0       -0.0502
      7 C          7.3818   -6.6509   -1.0790 C.2      0  UNK0        0.2068
      8 C          8.6947   -5.9698   -1.1044 C.2      0  UNK0        0.1126
      9 C          8.7747   -4.6289   -1.0109 C.2      0  UNK0        0.0978
     10 C          7.5420   -3.8157   -0.9025 C.2      0  UNK0        0.2089
     11 O          7.6167   -2.6111   -0.8211 O.2      0  UNK0       -0.2852
     12 O          7.3280   -7.8598   -1.0844 O.2      0  UNK0       -0.2856
     13 N         10.0040   -3.9888   -1.0179 N.pl3    1  ALA1       -0.3319
     14 CL        10.1351   -6.9011   -1.2398 Cl       0  UNK0       -0.0775
     15 CA        11.3033   -4.6775   -1.1428 C.3      1  ALA1        0.0575
     16 C         12.4070   -3.6468   -1.0854 C.2      1  ALA1        0.0616
```

```
    17 CB       11.4888   -5.6824    0.0000 C.3     1  ALA1       -0.0414
    18 O        13.6979   -4.0376   -0.9108 O.co2   1  ALA1       -0.5479
    19 OXT      12.1406   -2.4704   -1.1773 O.co2   1  ALA1       -0.5479
    20 H         2.9634   -3.7743   -0.8174 H       0  UNK0        0.0618
    21 H         2.8244   -6.1808   -0.9963 H       0  UNK0        0.0618
    22 H         4.8448   -7.5045   -1.1208 H       0  UNK0        0.0625
    23 H         5.1229   -2.6861   -0.7705 H       0  UNK0        0.0625
    24 H        10.0109   -3.0079   -0.9338 H       1  ALA1        0.1468
    25 HA       11.3344   -5.2058   -2.0727 H       1  ALA1        0.0556
    26 HB1      11.4597   -5.1664    0.9369 H       1  ALA1        0.0251
    27 HB2      12.4331   -6.1741   -0.1072 H       1  ALA1        0.0251
    28 HB3      10.7031   -6.4081   -0.0321 H       1  ALA1        0.0251
@<TRIPOS>UNITY_ATOM_ATTR
18 1
charge -1
```

Figure S3. Chemical structure of **NNQCl** and its optimized coordinates.

Molecular structure:

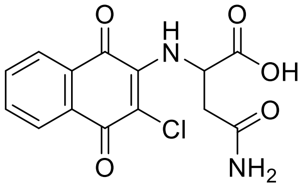

Optimized coordinates:

@<TRIPOS>MOLECULE

*****

32 33 0 0 0

SMALL

GASTEIGER

@<TRIPOS>ATOM

1	C	5.8533	-9.7927	-0.5601	C.ar	0	UNK0	-0.0610
2	C	5.7688	-11.1757	-0.6063	C.ar	0	UNK0	-0.0610
3	C	6.9233	-11.9417	-0.6344	C.ar	0	UNK0	-0.0502
4	C	8.1619	-11.3226	-0.6142	C.ar	0	UNK0	0.0340
5	C	8.2454	-9.9386	-0.5737	C.ar	0	UNK0	0.0344
6	C	7.0922	-9.1729	-0.5439	C.ar	0	UNK0	-0.0502
7	C	9.3974	-12.1276	-0.6269	C.2	0	UNK0	0.2068
8	C	10.7080	-11.4517	-0.7174	C.2	0	UNK0	0.1126
9	C	10.7932	-10.1081	-0.6667	C.2	0	UNK0	0.0979
10	C	9.5653	-9.2854	-0.5687	C.2	0	UNK0	0.2089
11	O	9.6417	-8.0782	-0.5236	O.2	0	UNK0	-0.2852
12	O	9.3401	-13.3329	-0.5293	O.2	0	UNK0	-0.2856
13	N	12.0229	-9.4699	-0.7131	N.pl3	1	ASN1	-0.3312
14	CL	12.1429	-12.3870	-0.8714	Cl	0	UNK0	-0.0775
15	CA	13.3300	-10.1222	-0.9255	C.3	1	ASN1	0.0685
16	C	14.4245	-9.1186	-0.6403	C.2	1	ASN1	0.0623
17	CB	13.5143	-11.3290	0.0000	C.3	1	ASN1	0.0473
18	O	15.6889	-9.5479	-0.3812	O.co2	1	ASN1	-0.5479
19	OXT	14.1762	-7.9350	-0.6257	O.co2	1	ASN1	-0.5479
20	CG	14.7630	-12.0605	-0.4200	C.2	1	ASN1	0.2126

```
21 OD1      15.7787  -11.4414   -0.6414 O.2      1  ASN1      -0.2760
22 ND2      14.7361  -13.4288   -0.5793 N.am     1  ASN1      -0.3293
23 H         4.9923   -9.2242   -0.5377 H        0  UNK0       0.0618
24 H         4.8449  -11.6354   -0.6198 H        0  UNK0       0.0618
25 H         6.8602  -12.9711   -0.6703 H        0  UNK0       0.0625
26 H         7.1554   -8.1434   -0.5097 H        0  UNK0       0.0625
27 H        12.0247   -8.4927   -0.5935 H        1  ASN1       0.1469
28 HA       13.3747  -10.4657   -1.9379 H        1  ASN1       0.0564
29 HB1      12.6689  -11.9799   -0.0814 H        1  ASN1       0.0380
30 HB2      13.6009  -11.0048    1.0160 H        1  ASN1       0.0380
31 HD21     13.9079  -13.9293   -0.3982 H        1  ASN1       0.1449
32 HD22     15.5451  -13.9060   -0.8742 H        1  ASN1       0.1449
@<TRIPOS>UNITY_ATOM_ATTR
18 1
charge -1
```

Figure S4. Chemical structure of **FNQCl** and its optimized coordinates.

Molecular structure:

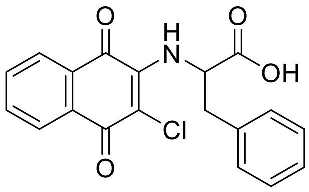

Optimized coordinates:

@<TRIPOS>MOLECULE

*****

38 40 0 0 0

SMALL

GASTEIGER

@<TRIPOS>ATOM

1	C	4.9633	-8.9770	-0.6101	C.ar	0	UNK0	-0.0610
2	C	4.8605	-10.3590	-0.6495	C.ar	0	UNK0	-0.0610
3	C	6.0051	-11.1392	-0.6814	C.ar	0	UNK0	-0.0502
4	C	7.2517	-10.5354	-0.6726	C.ar	0	UNK0	0.0340
5	C	7.3544	-9.1522	-0.6384	C.ar	0	UNK0	0.0344
6	C	6.2106	-8.3728	-0.6048	C.ar	0	UNK0	-0.0502
7	C	8.4783	-11.3561	-0.6951	C.2	0	UNK0	0.2068
8	C	9.8026	-10.7017	-0.8124	C.2	0	UNK0	0.1126
9	C	9.9048	-9.3589	-0.7701	C.2	0	UNK0	0.0979
10	C	8.6879	-8.5239	-0.6488	C.2	0	UNK0	0.2089
11	O	8.7909	-7.3203	-0.5842	O.2	0	UNK0	-0.2852
12	O	8.4024	-12.5595	-0.5853	O.2	0	UNK0	-0.2856
13	N	11.1358	-8.7229	-0.8290	N.pl3	1	PHE1	-0.3314
14	CL	11.2184	-11.6665	-0.9717	Cl	0	UNK0	-0.0775
15	CA	12.4651	-9.3451	-0.9861	C.3	1	PHE1	0.0642
16	C	13.5019	-8.2803	-0.7093	C.2	1	PHE1	0.0622
17	CB	12.6646	-10.5108	0.0000	C.3	1	PHE1	-0.0040

```
18 O       14.7843   -8.6426   -0.4262 O.co2  1  PHE1    -0.5479
19 OXT     13.1872   -7.1113   -0.7172 O.co2  1  PHE1    -0.5479
20 CG      13.9144  -11.3118   -0.3176 C.ar   1  PHE1    -0.0453
21 CD1     13.8298  -12.6879   -0.4493 C.ar   1  PHE1    -0.0585
22 CE1     14.9665  -13.4278   -0.7372 C.ar   1  PHE1    -0.0615
23 CZ      16.1874  -12.7921   -0.8956 C.ar   1  PHE1    -0.0617
24 CE2     16.2742  -11.4161   -0.7642 C.ar   1  PHE1    -0.0615
25 CD2     15.1381  -10.6781   -0.4757 C.ar   1  PHE1    -0.0585
26 H        4.1099   -8.3973   -0.5847 H      0  UNK0     0.0618
27 H        3.9308  -10.8070   -0.6551 H      0  UNK0     0.0618
28 H        5.9290  -12.1679   -0.7117 H      0  UNK0     0.0625
29 H        6.2866   -7.3440   -0.5759 H      0  UNK0     0.0625
30 H       11.1214   -7.7411   -0.7577 H      1  PHE1     0.1469
31 HA      12.5559   -9.7382   -1.9771 H      1  PHE1     0.0562
32 HB1     11.8150  -11.1590   -0.0539 H      1  PHE1     0.0335
33 HB2     12.7740  -10.1001    0.9820 H      1  PHE1     0.0335
34 HD1     12.9208  -13.1625   -0.3332 H      1  PHE1     0.0620
35 HE1     14.9031  -14.4533   -0.8336 H      1  PHE1     0.0618
36 HZ      17.0322  -13.3440   -1.1115 H      1  PHE1     0.0618
37 HE2     17.1832  -10.9417   -0.8810 H      1  PHE1     0.0618
38 HD2     15.2023   -9.6527   -0.3780 H      1  PHE1     0.0620
@<TRIPOS>UNITY_ATOM_ATTR
18 1
charge -1
```

Figure S5. Chemical structure of **GNQCl** and its optimized coordinates.

Molecular structure:

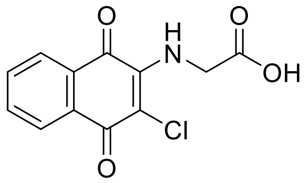

Optimized coordinates:

@<TRIPOS>MOLECULE

*****

25 26 0 0 0

SMALL

GASTEIGER

@<TRIPOS>ATOM

1	C	2.0681	-4.4303	-0.1035	C.ar	1	UNL1	-0.0610
2	C	2.0326	-5.8159	-0.1154	C.ar	1	UNL1	-0.0610
3	C	3.2139	-6.5404	-0.1184	C.ar	1	UNL1	-0.0502
4	C	4.4299	-5.8769	-0.1094	C.ar	1	UNL1	0.0340
5	C	4.4671	-4.4904	-0.1021	C.ar	1	UNL1	0.0344
6	C	3.2855	-3.7667	-0.0967	C.ar	1	UNL1	-0.0502
7	C	5.6901	-6.6408	-0.1009	C.2	1	UNL1	0.2068
8	C	6.9831	-5.9272	-0.1123	C.2	1	UNL1	0.1126
9	C	7.0266	-4.5812	-0.1085	C.2	1	UNL1	0.0976
10	C	5.7713	-3.7961	-0.1090	C.2	1	UNL1	0.2089
11	O	5.8155	-2.5876	-0.1393	O.2	1	UNL1	-0.2852
12	O	5.6671	-7.8500	-0.0650	O.2	1	UNL1	-0.2856
13	N	8.2431	-3.9195	-0.1113	N.pl3	1	UNL1	-0.3344
14	CL	8.4485	-6.8272	-0.1233	Cl	1	UNL1	-0.0775
15	C	9.5213	-4.6510	-0.1158	C.3	1	UNL1	0.0489
16	C	10.6796	-3.6858	-0.0876	C.2	1	UNL1	0.0587
17	O	11.9532	-4.1592	-0.1403	O.co2	1	UNL1	-0.5482
18	O	10.4770	-2.4962	0.0000	O.co2	1	UNL1	-0.5482
19	H	1.1876	-3.8921	-0.0997	H	1	UNL1	0.0618

20	H	1.1257	-6.3084	-0.1220	H	1	UNL1	0.0618
21	H	3.1878	-7.5720	-0.1273	H	1	UNL1	0.0625
22	H	3.3115	-2.7351	-0.0876	H	1	UNL1	0.0625
23	H	8.2537	-2.9351	-0.1103	H	1	UNL1	0.1466
24	H	9.5822	-5.2490	-1.0010	H	1	UNL1	0.0522
25	H	9.5676	-5.2741	0.7528	H	1	UNL1	0.0522

@<TRIPOS>UNITY_ATOM_ATTR

17 1

charge -1

Figure S6. Chemical structure of **MNQCl** and its optimized coordinates.

Molecular structure:

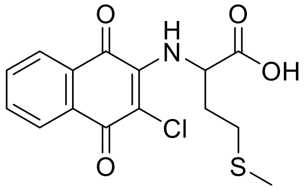

Optimized coordinates:

@<TRIPOS>MOLECULE

*****

35 36 0 0 0

SMALL

GASTEIGER

@<TRIPOS>ATOM

1	C	4.1083	-7.6616	-0.5065	C.ar	0	UNK0	-0.0610
2	C	4.0355	-9.0448	-0.5647	C.ar	0	UNK0	-0.0610
3	C	5.1974	-9.7997	-0.5997	C.ar	0	UNK0	-0.0502
4	C	6.4331	-9.1709	-0.5741	C.ar	0	UNK0	0.0340
5	C	6.5052	-7.7863	-0.5197	C.ar	0	UNK0	0.0344
6	C	5.3434	-7.0320	-0.4841	C.ar	0	UNK0	-0.0502
7	C	7.6788	-9.9626	-0.6010	C.2	0	UNK0	0.2068
8	C	8.9835	-9.2736	-0.6950	C.2	0	UNK0	0.1126
9	C	9.0605	-7.9308	-0.6352	C.2	0	UNK0	0.0978
10	C	7.8259	-7.1232	-0.5056	C.2	0	UNK0	0.2089
11	O	7.9014	-5.9187	-0.4174	O.2	0	UNK0	-0.2852
12	O	7.6393	-11.1694	-0.5178	O.2	0	UNK0	-0.2856
13	N	10.2835	-7.2816	-0.6983	N.pl3	1	MET1	-0.3316
14	CL	10.4254	-10.1999	-0.8591	Cl	0	UNK0	-0.0775
15	CA	11.5879	-7.9195	-0.9725	C.3	1	MET1	0.0610
16	C	12.6841	-6.9526	-0.5908	C.2	1	MET1	0.0619
17	CB	11.7695	-9.2231	-0.1811	C.3	1	MET1	-0.0203

```
    18 O        13.9286   -7.4136   -0.2964 O.co2   1  MET1       -0.5479
    19 OXT     12.4528   -5.7667   -0.5382 O.co2   1  MET1       -0.5479
    20 CG      12.9993   -9.9727   -0.7002 C.3      1  MET1       -0.0046
    21 SD      13.0170  -11.6405    0.0000 S.3      1  MET1       -0.1641
    22 CE      14.5738  -12.3510   -0.5894 C.3      1  MET1       -0.0181
    23 H         3.2427   -7.1003   -0.4798 H        0  UNK0        0.0618
    24 H         3.1158   -9.5126   -0.5820 H        0  UNK0        0.0618
    25 H         5.1429  -10.8293   -0.6448 H        0  UNK0        0.0625
    26 H         5.3974   -6.0023   -0.4409 H        0  UNK0        0.0625
    27 H       10.2831   -6.3088   -0.5469 H        1  MET1        0.1469
    28 HA      11.6303   -8.1629   -2.0136 H        1  MET1        0.0559
    29 HB1    10.9013   -9.8368   -0.3019 H        1  MET1        0.0295
    30 HB2     11.9048   -8.9935    0.8552 H       1  MET1        0.0295
    31 HG1     13.8866   -9.4517   -0.4066 H      1  MET1        0.0377
    32 HG2     12.9612  -10.0310   -1.7679 H      1  MET1        0.0377
    33 HE1     14.6722  -13.3504   -0.2201 H      1  MET1        0.0340
    34 HE2     15.3922  -11.7585   -0.2372 H      1  MET1        0.0340
    35 HE3     14.5774  -12.3642   -1.6593 H      1  MET1        0.0340
@<TRIPOS>UNITY_ATOM_ATTR
18 1
charge -1
```

2. Ligand-receptor interactions.

All the top ligand-receptor interactions were visualized using Discovery Studio. Here, we present the 2D interaction diagrams, while the manuscript includes only the 3D representations of the best ligand-receptor interactions.

Table S1. Summary of principal ligand–receptor interactions identified for the docked **NQ-derived** compounds.

**Ligand**	**Interaction type**	**Receptor residue**
NNQ	Hydrogen bond	Arg B:248
		Ser A:241
		Guanine E:12
ANQCl	Hydrogen bond	Adenine E:6
		Arg B:248
		Guanine E:8
	Pi-alkyl	Guanine F:8
NNQCl	Hydrogen bond	Ser A:241
		Arg B:248
		Guanine E:8
		Guanine E:12
FNQCl	Hydrogen bond	Met A:243
		Guanine E:12
	Pi-sulphur	Cys B:242
		Met A:243
GNQCl	Hydrogen bond	Arg B:248
		Guanine E:8
		Guanine E:12
MNQCl	Hydrogen bond	Arg B:248
		Guanine E:12
	Alkyl	Met A:243

Figure S7. Two-dimensional interaction diagrams of the **NNQ–p53**, **ANQCl–p53**, **NNQCl–p53**, **FNQCl–p53**, **GNQCl–p53**, and **MNQCl–p53** complexes.

NNQ-p53:

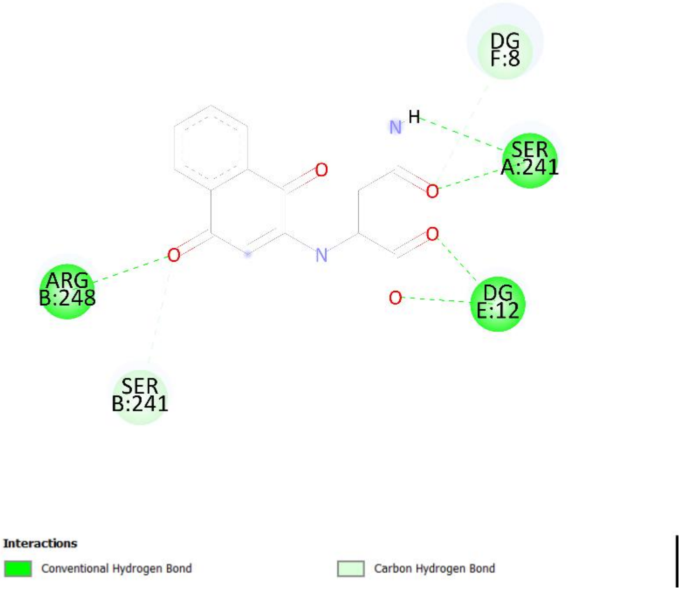

ANQCl-p53:

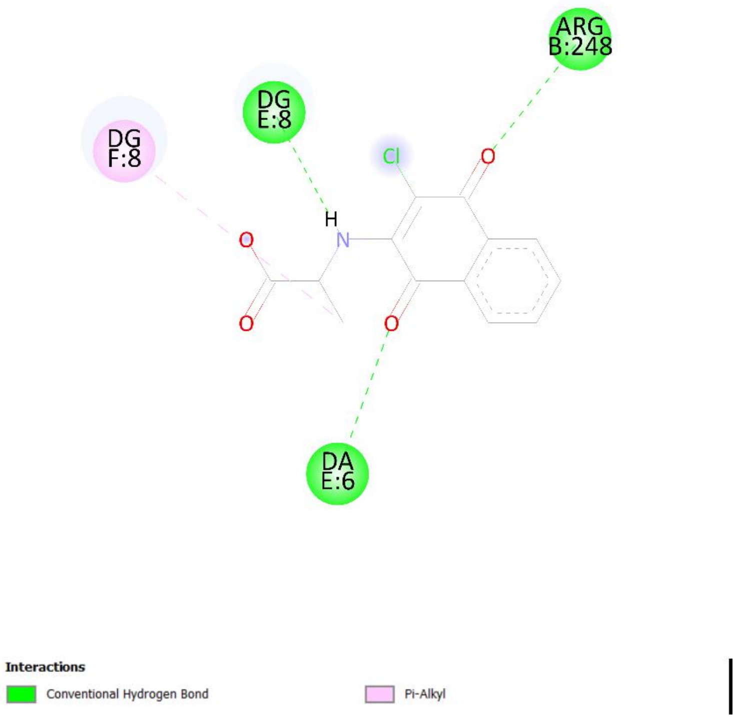

NNQCl-p53:

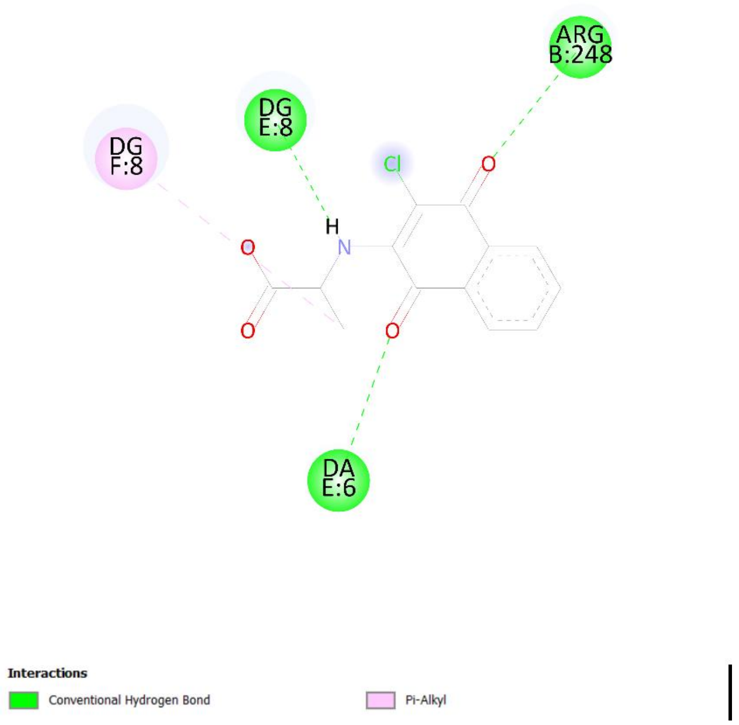

FNQCl-p53:

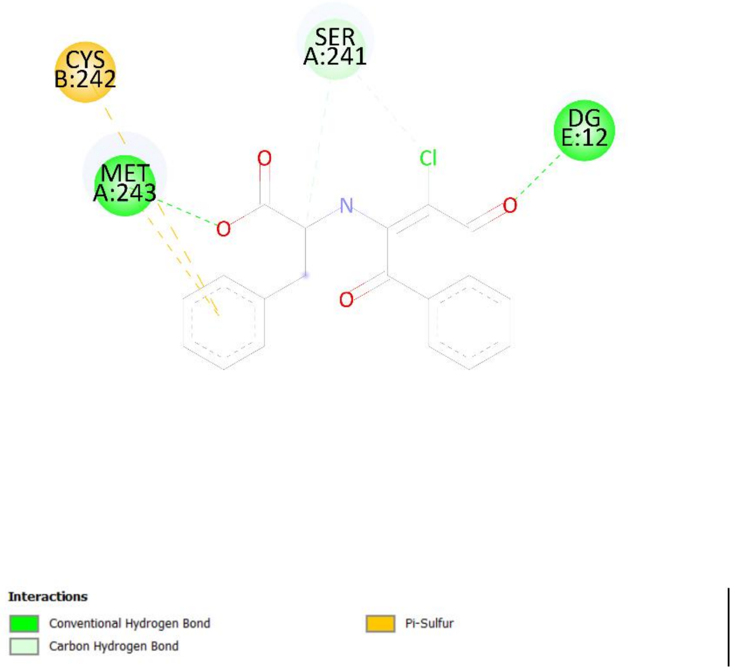

GNQCl-p53:

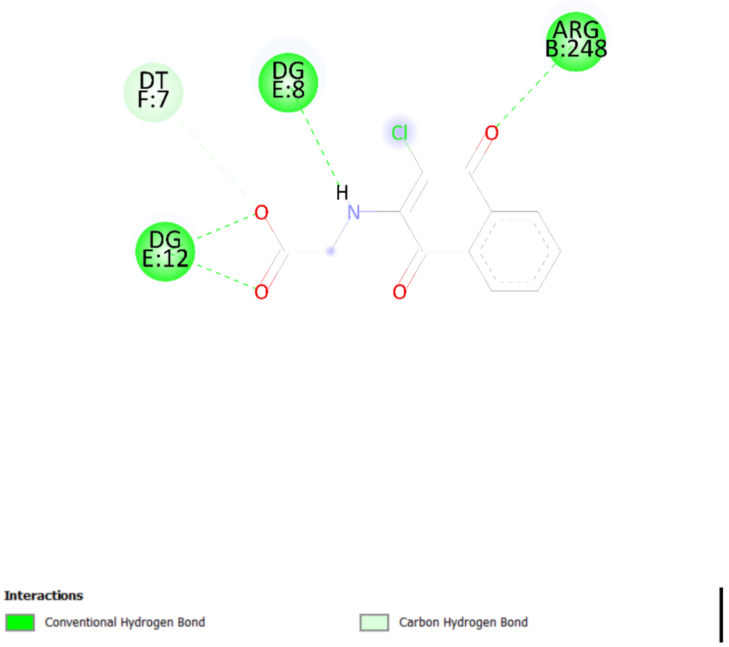

MNQCl-p53:

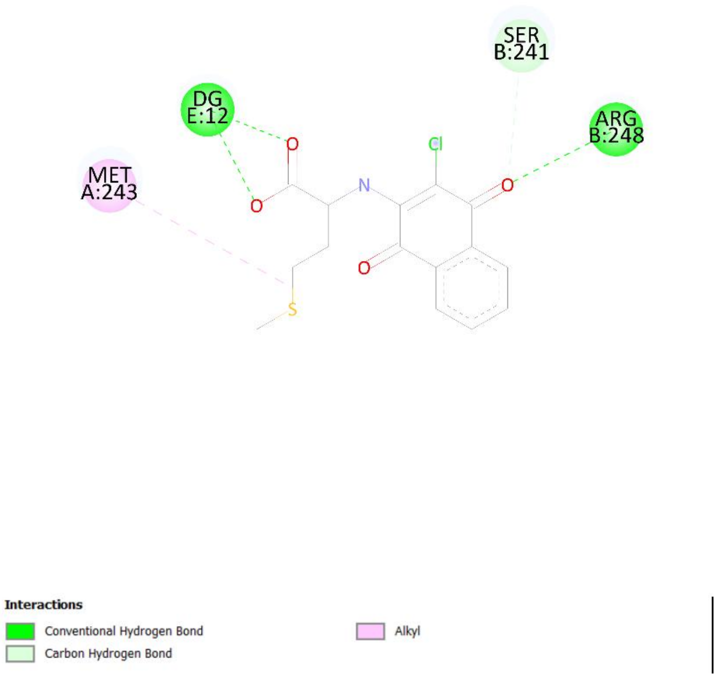

Supplement: Supplementary file 1 [file ijms-27-05703-s001.zip › ijms-4338478-supplementary.pdf]
